# ERK2-Mediated Phosphorylation of Transcriptional Coactivator Binding Protein PIMT/NCoA6IP at Ser^298^ Augments Hepatic Gluconeogenesis

**DOI:** 10.1371/journal.pone.0083787

**Published:** 2013-12-17

**Authors:** Bandish Kapadia, Navin Viswakarma, Kishore V. L. Parsa, Vasundhara Kain, Soma Behera, Sashidhara Kaimal Suraj, Phanithi Prakash Babu, Anand Kar, Sunanda Panda, Yi-jun Zhu, Yuzhi Jia, Bayar Thimmapaya, Janardan K. Reddy, Parimal Misra

**Affiliations:** 1 Department of Biology, Dr Reddy’s Institute of Life Sciences, An Associate Institute of University of Hyderabad, Hyderabad, Andhra Pradesh, India; 2 Department of Biotechnology, School of Life Sciences, University of Hyderabad, Hyderabad, Andhra Pradesh, India; 3 Department of Life Sciences, Devi Ahilya University, Indore, Madhya Pradesh, India; 4 Department of Pathology, Feinberg School of Medicine, Northwestern University, Chicago, Illinois, United States of America; 5 Department of Microbiology and Immunology, Feinberg School of Medicine, Northwestern University, Chicago, Illinois, United States of America; Kobe University, Japan

## Abstract

**P**RIP-**I**nteracting protein with **m**ethyl **t**ransferase domain (PIMT) serves as a molecular bridge between CREB-binding protein (CBP)/ E1A binding protein p300 (Ep300) -anchored histone acetyl transferase and the Mediator complex sub-unit1 (Med1) and modulates nuclear receptor transcription. Here, we report that ERK2 phosphorylates PIMT at Ser^298^ and enhances its ability to activate PEPCK promoter. We observed that PIMT is recruited to PEPCK promoter and adenoviral-mediated over-expression of PIMT in rat primary hepatocytes up-regulated expression of gluconeogenic genes including PEPCK. Reporter experiments with phosphomimetic PIMT mutant (PIMT^S298D^) suggested that conformational change may play an important role in PIMT-dependent PEPCK promoter activity. Overexpression of PIMT and Med1 together augmented hepatic glucose output in an additive manner. Importantly, expression of gluconeogenic genes and hepatic glucose output were suppressed in isolated liver specific PIMT knockout mouse hepatocytes. Furthermore, consistent with reporter experiments, PIMT^S298D^ but not PIMT^S298A^ augmented hepatic glucose output via up-regulating the expression of gluconeogenic genes. Pharmacological blockade of MAPK/ERK pathway using U0126, abolished PIMT/Med1-dependent gluconeogenic program leading to reduced hepatic glucose output. Further, systemic administration of T_4_ hormone to rats activated ERK1/2 resulting in enhanced PIMT ser^298^ phosphorylation. Phosphorylation of PIMT led to its increased binding to the PEPCK promoter, increased PEPCK expression and induction of gluconeogenesis in liver. Thus, ERK2-mediated phosphorylation of PIMT at Ser^298^ is essential in hepatic gluconeogenesis, demonstrating an important role of PIMT in the pathogenesis of hyperglycemia.

## Introduction

PIMT/NCoA6IP (**P**RIP **I**nteracting protein with Methyl Transferase domain) was first isolated as a transcriptional coactivator PRIP/NCoA6 interacting protein (NCoA6IP) in a yeast two hybrid screen [1]. PIMT is expressed in several tissues including liver, kidney and skeletal muscle [1]. PIMT is expressed ubiquitously and contains an RNA binding motif, a putative methyltransferase domain and an S-adenosyl methionine (SAM) binding domain, suggesting that it may function as an RNA methyltransferase[1]. Later it was demonstrated that PIMT hypermethylates small nuclear RNAs (snRNAs) and small nucleolar RNAs (snoRNAs) [2]. DTL, the *Drosophila* homolog of PIMT/NCoA6IP has an essential role in development [3]. Recently, we reported that disruption of PIMT gene results in early embryonic lethality around E3.5 days due to impairment of development affecting the blastocyst and uterine implantation stages. These findings establish that PIMT/NCoA6IP which is expressed in all cells of the E3.5 day stage blastocyst is indispensable for early embryonic development [4]. 

PIMT interacts with and co-localizes to nucleus along with histone acetyl transferase (HAT) containing transcriptional coactivators such as CBP/Ep300 and non-HAT containing coactivators like the Mediator subunit Med1 (PPAR binding protein, PBP/TRAP220/DRIP205) and PRIP [1,5,6].Therefore, we proposed that PIMT serves as a molecular bridge between HAT and non-HAT containing transcriptional complexes and controls nuclear receptor-mediated transcription. PIMT enhances Med1 and PRIP-mediated transcription whereas it inhibits CBP/Ep300 mediated transcription, raising the possibility that it contextually modulate transcriptional machinery [6]. It is widely regarded that regulation of some of the nuclear receptor co-regulators is modulated *via* post-translational modifications, importantly by phosphorylation [7-9]. Indeed, stimulation of CBP/Ep300 or SRC-1 dependent transcriptional activity by MAPK signaling elucidates that phosphorylation exerts a positive regulatory effect on certain coactivator functions [10-15]. In contrast, phosphorylation of Ep300 by protein kinase C (PKC) has been shown to attenuate its transcriptional activity suggesting that different signaling pathways differentially regulate the function of coactivators [16]. We and others previously demonstrated that Med1 is a substrate of protein kinase A (PKA), PKC, and ERK2/ERK5 and that ERK2-mediated modification of Med1 enhances its coactivator function [17,18].Because very little information is available about the mechanisms that influence the function of coactivator binding protein PIMT, we sought to examine the influence of phosphorylation of PIMT in controlling its function.

PEPCK, a PPAR regulated gene, is an important regulator of gluconeogenesis [19]. Gluconeogenesis, mainly occurring in liver, is a process through which *de novo* synthesis of glucose occurs from non-carbohydrate precursors such as lactate, pyruvate, glycerol and alanine. PEPCK expression is powerfully controlled at the transcriptional level by key hormones, particularly insulin, glucagon and glucocorticoids. PEPCK promoter contains several *cis*-regulatory responsive elements like peroxisome proliferator responsive element(PPRE), glucocorticoid response element (GRE), thyroid hormone response element (TRE) and cAMP response element where different transcription factors bind to and modulate the gene expression [20]. Additionally, co-activators such as CBP/Ep300, transducers of regulated CREB protein 2 (TORC2) and peroxisome proliferator activated receptor gamma coactivator 1 alpha (PGC1α) also regulate PEPCK expression in liver [21].

In this study, we show for the first time that ERK2 phosphorylates PIMT at Ser^298^ which increases Med1-PPARγ mediated transcriptional activity. Further, we show that PIMT or Med1-dependent PEPCK promoter activity was sensitive to phosphorylation by RAF-BXB/ERK2. Presence of negative charge on Ser^298^ of PIMT enhances its ability to augment hepatic gluconeogenesis. PIMT was recruited to PEPCK promoter and a significant increase in the PEPCK expression was observed in liver upon adenoviral-mediated overexpression of PIMT. We further demonstrated that PIMT and Med1 exert an additive and MAPK/ERK-dependent inductive effect on hepatic glucose output. Importantly, we also show that deficiency of PIMT resulted in the reduction of PEPCK expression and hepatic glucose output. Finally, we have shown that systemic administration of T_4_ hormone to rats activated MEK/ERK resulting in PIMT Ser^298^ phosphorylation. Phosphorylation of PIMT by this mechanism led to its increased binding to the PEPCK promoter, increased PEPCK expression and induction of gluconeogenesis in liver confirming that PIMT is phosphorylated in intact liver cells and this pathway is functional in the liver at physiological levels of PIMT and PEPCK*.*

## Experimental Procedures

### Ethics statement

Colony bred healthy adult Wister albino rats, weighing 150±10g were maintained in polypropylene cages in a standard photoperiod (12 h light:12 h dark cycle) and temperature (27 ± 1°C) controlled room with the provision of laboratory food (Gold Mohur feeds Ltd, New Delhi, India) and water *ad libitum*. The experiment was carried out according to the guidelines of the Committee for the Purpose of Control and Supervision on Experiments on Animals (CPCSEA), Experimental protocol involving animals was reviewed and approved by the Animal Ethical Committee of our University, Devi Ahilya Viswavidyalaya, Indore, India (Regd. No.799). All animal procedures used in PIMT knockout study were reviewed and pre- approved by the Institutional Review Boards for Animal Research of the Northwestern University (protocol number 2012-1519).

### Expression vectors and promoter constructs

Expression plasmids pcDNA3.1-PPARγ, pcDNA3.1-Med1, pCMX-Med1, pcDNA3.1-PIMT, pcDNA3.1-PIMT-N (1-334), pCMV-PIMT-Flag, 3XPPRE-Luc, GST-PIMT-N (1-334), GST-Med1-C (1371-1560), RAF-BXB and pCMV-ERK2-HA were described previously [6,17]. Genomic DNA of HEK293 cells encompassing ~ 2kb region upstream of PEPCK transcriptional start site was amplified and inserted into *SacI/XhoI* sites of reporter vector pGL3-Basic (Promega, Madison, WI, USA). Primer sequences are provided in supporting information (Table S1). PIMT^S298A^, PIMT^S298D^ and GST-PIMT-M (1-334, Ser^298^Ala) mutant were generated by site directed mutagenesis kit (Agilent technologies, Palo Alto, CA, USA) according to manufacturer’s recommendations.

### Cell culture and transfection

HeLa and HEK293T were maintained in medium containing DMEM with 10% fetal bovine serum at 37°C with 5% CO_2_ environment. For transactivation assays, cells were transfected with thymidine kinase promoter containing 3XPPRE driven luciferase construct, along with other DNA as mentioned. For PEPCK promoter activity studies, 293T cells were transfected with pGL3-PEPCK full length promoter in addition to other DNA constructs mentioned. All transfections were carried out using Lipofectamine 2000 (Invitrogen, Carlsbad, CA, USA) according to manufacturer’s instructions. Each transfection mix contained 700ng of reporter plasmid DNA, 100ng of PPARγ, 350ng of Med1 and 300ng of β-galactosidase/ renilla luciferase expression vectors along with 350ng of PIMT or PIMT^S298A^ or PIMT^S298D^expression construct. Where indicated, 350ng of each RAF-BXB and ERK2 were also co-transfected. Cells were lysed 30h after transfection with reporter lysis buffer (for beta galactosidase assay) or passive lysis buffer (for renilla luciferase) (Promega, Madison, WI, USA) and equal amounts of lysate and luciferase assay buffer (20mM tricine, 1.07mM magnesium carbonate, decahydrate, 0.1mM EDTA, 2.67mM MgSO4, 33.3mM DTT, 270µM co-enzyme A, 470µM d-Luciferin, 530µM ATP) were mixed and luminescence values were recorded in Sirius Luminometer (Berthold Detection Systems GmbH, Pforzheim, Baden-Württemberg, Germany). The values were normalized to the activity from co-transfected β-galactosidase expression vector (3) or renilla luciferase activity (renilla luciferase assay buffer: 4µM coelentrazine, 50µM phenyl benzothiazole, 25mM sodium pyrophosphate, 15mM EDTA, 10mM sodium acetate, 500mM sodium sulphate and 500mM sodium chloride).

### In vitro and cellular phosphorylation of PIMT

In vitro kinase reaction with GST-PIMT fragments were carried out as reported earlier[17]. For cellular phosphorylation assay, 293T cells were transfected with 16µg of pCMV-PIMT Flag or pCMV- PIMT^S298A^ Flag along with RAF-BXB using Lipofectamine 2000 (Invitrogen, Carlsbad, CA, USA). Post transfection, cells were cultured in DMEM containing 1% FBS overnight (to reduce background phosphorylation). Cells were lysed in TENN lysis buffer (20mM Tris-HCl pH 8.0, 1mM EDTA, 100mM sodium chloride, 0.5 % NP-40, 10mM sodium pyrophosphate 10mM sodium fluoride and 10mM sodium orthovandate, 10µg of aprotinin and 10µg of Leupeptin). PIMT was immunoprecipitated using Anti-Flag magnetic beads (Sigma, St. Louis, MO, USA) or Anti-PIMT bound to protein G magnetic beads. After overnight incubation at 4°C, the beads were washed four times with lysis buffer and subsequently were reconstituted in Lamelli lysis buffer and separated on 10% SDS-PAGE. The separated proteins were transferred to PVDF membrane and probed by Anti-MPM2 (pSer-Pro; ERK substrate antibody, 1µg/mL) (Millipore, Bedford, MA USA) followed by Anti-Flag (1:2000, Sigma, St. Louis, MO, USA). Nonspecific Anti-rabbit IgG or Anti-goat IgG (Santacruz Biotechnology Inc., Santacruz, CA, USA) was used as the negative control.

### Chromatin Immunoprecipitation

The protocol was adopted from the methods described earlier with minor modifications [22]. HepG2 cells (~15 millions) were incubated on shaker with formaldehyde (1.5%) for a period of 10 min for fixing. To arrest the cross linking, glycine (125mM) was added directly on the plate and incubated on shaker for 10 min. The cells were scrapped and were washed twice with ice cold PBS (containing 1mM PMSF). The cells were lysed using SDS lysis buffer (1%SDS, 10mM EDTA, 50mM Tris-HCl pH 8.0, 1µg/ml aprotinin, 1µg/ml leupeptin and 1mM PMSF) and incubated on ice for 15 min. To shear the chromatin, the lysate was sonicated using Vibra cell sonicator (Sonics and Material Inc., Newton, CT, USA) [10 sec pulse on, 10 sec pulse off and amplitude 30 sec] on ice yielding a chromatin fragment of ~500bp in size. Samples were centrifuged at 14,000 rpm for 10 min and supernatant was divided in aliquots (200µl) for 10 fold dilution in ChIP dilution buffer (0.01% SDS, 1.1% Triton X-100, 1.2mM EDTA, 16.7 mMTris-HCl pH 8.0, 167 mM NaCl, 1µg/ml aprotinin, 1µg/ml leupeptin and 1mM PMSF). To have a positive control, 50µL aliquot was stored for further processing with the other samples at the reverse cross linking step. To reduce the non-specific background the diluted lysates were incubated with 20µL ofofBSA blocked magnetic protein G beads (GenScript, Piscataway,NJ, USA) for 1h at 4°C. The beads were removed and the pre-cleared lysate was incubated either with PIMT Antibody (2µg, Abcam, Cambridge, UK) or non-specific Rabbit IgG (Santacruz Biotechnology Inc., Santacruz, CA, USA) for immunoprecipitation chromatin complex at 4°C overnight. The chromatin was pulled down using salmon sperm DNA blocked magnetic Protein G beads (20µL, GenScript, Piscataway, NJ USA) while rotating at 4°C for 2h. The beads were washed successively with the following buffers for 5 min at 4°C with 1mL: Low salt buffer (0.1% SDS, 1% Triton X-100, 2mM EDTA, 20mM Tris-HCl pH 8.0, 150mM NaCl), High salt buffer (0.1% SDS, 1% Triton X-100, 2mM EDTA, 20mM Tris-HCl pH 8.0, 500mM NaCl), LiCl wash buffer (0.25mM LiCl, 1 % NP-40, 1% sodium deoxycholeate, 1mM EDTA and 10mM Tris-HCl pH 8.0) and with TE buffer (10mM Tris-HCl pH8.0 and 1mM EDTA). Chromatin complexes were eluted from the beads in two consecutive washes with 250µL of elution buffer (1% SDS and 0.1M sodium bicarbonate) for 15 min at room temperature. The reverse cross linking was performed using 200mM sodium chloride for 65°C for 4h. The protein was digested using Proteinase K treatment (20µg in 10mM EDTA, 40mM Tris-HCl pH 6.5). Chromatin was purified using phenol:cholorform:isoamylalchol (25:24:1) and the DNA was precipitated using 100% chilled ethanol and 1/10 volume of sodium acetate followed by washing twice with 70% ethanol. Samples were resuspended in 30µL of TE (10mM Tris-HCl pH8.0 and 1mM EDTA) and stored at -20°C for subsequent PCR analysis.

### Adenovirus generation, northern blotting and qPCR

Ad/Med1 and Ad/eGFP were generated as described previously [10,23] and Ad/LacZ was a kind gift from W. El-Deiry, University of Pennsylvania, Philadelphia, USA. Ad/PIMT eGFP was constructed using full-length human PIMT cloned into peGFP-C1 vector (Clontech, Palo Alto, CA, USA) to generate PIMT eGFP fused DNA. PIMT eGFP was then retrieved and cloned into pShuttle-CMV expression vector (Quantum Biotechnologies Inc., Montreal, Quebec, Canada) at EcoRV sites. PIMT^S298A^ and PIMT^S298D^ were cloned into pShuttle-CMV expression vector (Quantum Biotechnologies Inc., Montreal, Quebec, Canada) at *BglII/XhoI* sites. The linearized shuttle vector and AdEasy vector (Quantum Biotechnologies Inc., Montreal, Quebec, Canada) were then co-transformed into Escherichia coli strain BJ5183. Positive recombinant plasmid was selected and virus was then generated as described for Ad/Med1 virus (5).A replication incompetent recombinant adenovirus containing PIMT (Ad/PIMT eGFP) or Med1 (Ad/Med1) and LacZ (Ad/LacZ) were injected intravenously into the tail vein of wild-type (C57BL/6J; 3-4 weeks old) mice in a volume of 200μl containing 1 × 10^11^ virus particles and were sacrificed after 3 or 5 days. Ad/LacZ injection was used as a control. Livers were harvested; snap frozen in liquid nitrogen and total RNA isolated using Trizol reagent (Invitrogen, Carlsbad, CA, USA). Twenty micrograms of RNA was electrophoresed, transferred to nylon membrane and was hybridized to ^α32^P radio labeled probe and signals were visualized by autoradiography. For qPCR, reverse transcription was performed with 4μg of total RNA using random hexamer in a reaction volume of 20μl using Superscript III First Strand cDNA Synthesis System (Invitrogen, Carlsbad, CA, USA). Quantitative PCR for PEPCK expression and other gluconeogenic genes was performed using SYBR green master mix (TaKaRa, Otsu, Shinga, Japan) on master cycler ep realplex 4 (Eppendorf, Hamburg, Germany). The mRNA expression was normalized with the reference genes. All experiments were repeated three times. 

### Generation of liver specific PIMT Knockout mice

Generation of PIMT^fl/fl^ was described previously [4]. Liver specific PIMT knockout (PIMT^ΔLivKO^ mice) was generated by crossing PIMT^fl/fl^ mice with albumin-cre transgenic mice as reported previously [24]. 

### Immunohistochemistry

Liver from fed or fasting mice were fixed in 4% paraformaldehyde in PBS by immersion, dehydrated and embedded in paraffin. Tissue blocks were cut into 5 µm-thick sections. Sections were dewaxed, washed once in absolute ethanol, and incubated in 3.3% hydrogen peroxide in methyl alcohol for 40 min in the dark at room temperature in order to inactivate endogenous peroxidase. The section were hydrated in graded alcohol followed by 3 washes in PBS for 5 min. Sections were incubated in blocking solution containing 5% BSA and 2% horse serum for 2 h, followed by overnight incubation at 4°C with Anti-PIMT antibody (diluted 1:350; Bethyl, Montgomery, TX, USA). Sections were then washed three times with PBS, incubated in a biotinylated goat anti-rabbit IgG (diluted 1:200) for 1 h, washed again in PBS, and incubated in Vectastain ABC kit (Vector Labs Inc., Burlingame, CA, USA) for 1 h. After three washes in PBS, immunolabeling was revealed with 0.05% diaminobenzidine (DAB). Sections were then dehydrated in ethanol, cleared in xylene, and coverslipped with mounting medium.

### Glucose production assay in primary hepatocytes

Hepatocytes were isolated from female Sprague Dawley (SD) rats by collagenase digestion [25] and were maintained in DMEM containing 10% FBS. Primary hepatocytes were seeded (0.5 x 10^6^) into collagen coated 12-well plates and were infected with Ad/PIMT, Ad/PIMT^S298A^, Ad/PIMT^S298D^, Ad/Med1 separately or together. Ad/eGFP or Ad/LacZ was used as the virus control. Medium was replaced 6h after infection with complete medium. Twelve-hours later medium was changed again with glucose estimation assay medium (phenol-red and glucose-free DMEM containing 20mM sodium lactate, 2mM sodium pyruvate). Cell supernatants were collected 6 h later and were subjected to glucose measurement using Glucose (GO) assay kit (GAG0-20, Sigma,St. Louis, MO, USA). Cells were lysed, and protein concentration was determined using Bradford reagent. The glucose output was normalized to cellular protein concentration and was expressed as arbitrary units.

### Animals, T_4_ stimulus and Serum and Liver Tissue Analysis

#### Induction of hyperglycemia with L-Thyroxine (L-T_4_)

Ten rats were divided in to two groups of five each. Initial body weight was recorded. Animals of group 1 receiving the vehicle, normal saline (0.1 mL/animal) served as control and those of group II were injected intra-peritoneal (0.1 mL/animal) with L-T_4_ for 2 weeks (1mg /kg /d) to induce hyperthyroidism[25]. 

#### Sample preparation

On the last day of experimentation, final body weight was taken and overnight fasted animals were sacrificed by cervical dislocation after exposing them to mild ethyl ether anesthesia. All efforts were made to minimize suffering. Blood samples were collected, allowed to clot and centrifuged to get a clear serum, which was stored at −20 °C until further estimations. Liver was removed quickly ,washed off blood clots and stored in liquid nitrogen for further use.

#### Assay of thyroid hormones

Total thyroid hormones levels were determined using RIA kits (Immunotech, Beckman Coulter, Czech Republic) as done routinely in our laboratory[26,27]. In brief, for RIA the anti-sera, specific hormone standards, radio labeled hormones (I^125^ T_4_ and I^125^ T_3_), and the control sera were reconstituted with assay buffer/double distilled water. The reaction mixture comprised of standard/sample, buffer, radio labeled hormone, and the respective antibody was incubated at 25°C (1 hour in shaker). After decanting the supernatant, the tubes were subjected to radioactivity counting for one minute (CPM) using an I^125^ gamma counter. A set of quality control sera of rat was also used with each assay. Intra- and inter assay variations for T_4_ were below 6.2% and 8.6% respectively and for and T_3_ 3.3%, and 8.6% respectively. 

### *In vivo* phosphorylation and Chromatin Immunoprecipitation assay

Liver tissue lysates were prepared in RIPA lysis buffer. PIMT was immunoprecipitated using Anti-PIMT (2 µg, N-13, Santacruz Biotechnology Inc., Santacruz, CA, USA), separated on 6% SDS PAGE and probed by Anti-MPM2 (ERK substrate antibody, 1µg/mL) (Millipore, Bedford, MA USA) followed by Anti-PIMT(1:1000, N-13, Santacruz Biotechnology Inc., Santacruz, CA, USA). ChIP was performed as mentioned above using Anti-PIMT (2 µg, N-13, Santacruz Biotechnology Inc., Santacruz, CA, USA).

### Western blot analysis

Proteins were extracted from primary hepatocytes and liver tissue and subjected to SDS-PAGE and transferred onto PVDF membrane. Western analysis was performed with Anti-GFP, Anti-pERK (1:1000), total ERK antibodies (1:1000), Anti- PEPCK (Santacruz Biotechnology Inc., Santacruz, CA, USA) and Anti-Beta actin (Cell Signaling Technology, Cambridge, MA, USA) and detected by ECL reagent (Amersham Biosciences, Piscataway, NJ, USA). 

### Statistical analysis

Values were expressed as mean ± S.D. For comparison between 2 groups, the unpaired Student’s *t*-test was used. One-way ANOVA followed by Bonferroeni’s post hoc analysis was used to compare more than 2 groups. *p*< 0.05 was considered as significant.

## Results

### PIMT is a substrate of ERK2

Previously, we reported that PIMT and Med1 interact with each other and that transcriptional activity of Med1 is enhanced through ERK2-mediated phosphorylation [6,17]. Thus, to test whether the function of PIMT is also regulated by post translational modifications like phosphorylation, we performed computational analysis to identify potential phosphorylation sites on PIMT. Such analysis revealed the presence of one MAPK/ERK consensus site of phosphorylation at Ser^298^[^296^PS**S**P]. In addition to the conserved phosphorylation site, PIMT also contained putative MAPK docking sites; which frequently mediate high level interaction between MAPK kinase and its substrate. PIMT has one D domain (^119^ **RNKVKIKKK**) and one DEF motif (^228^ **WNFP**; Figure S1) [23,26-31]. These observations led us to examine whether PIMT is a substrate of MAPK/ERK kinase.

**Figure 1:**
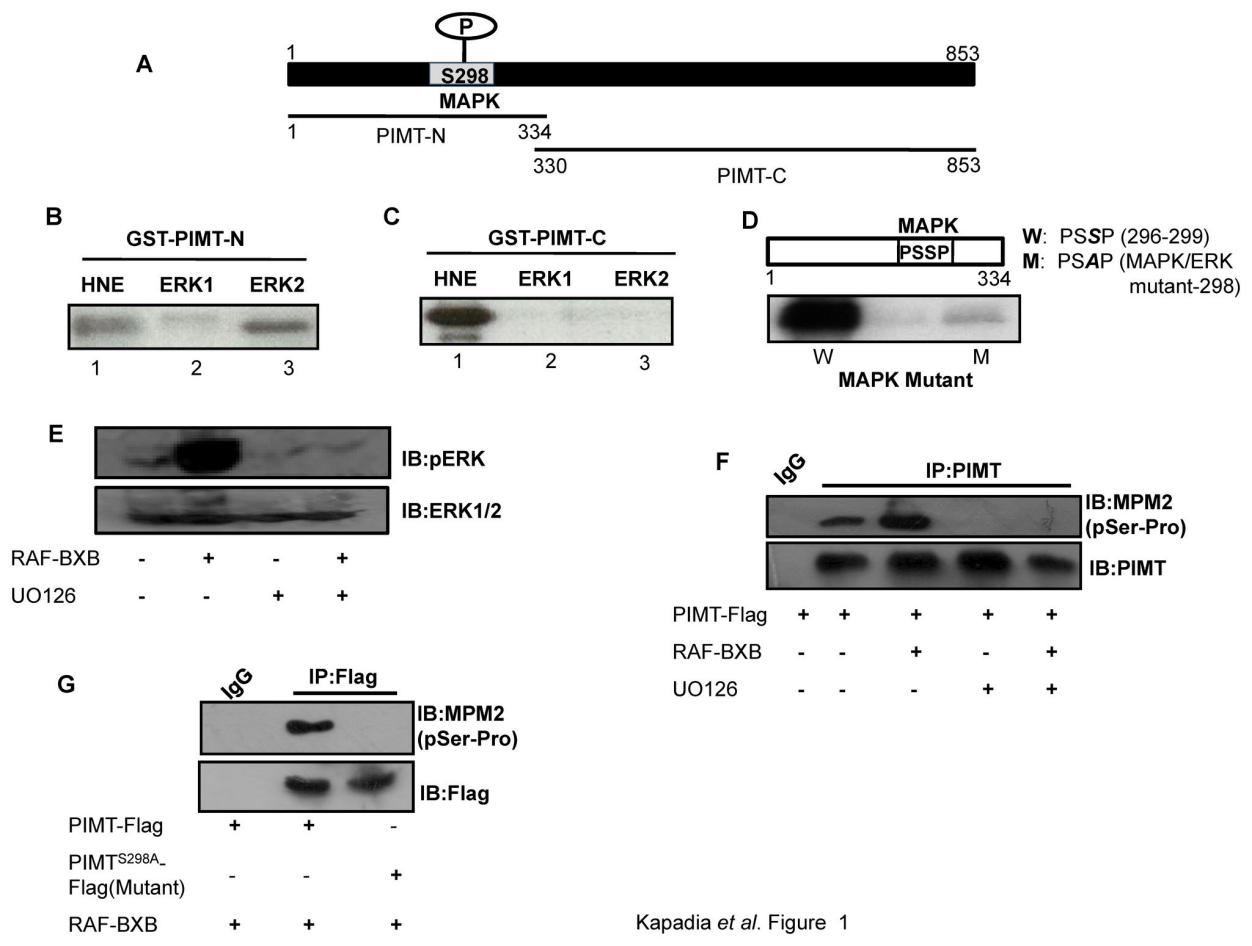
PIMT is a substrate of MAPK. (**A**) Schematic diagram of PIMT with a potential phosphorylation site of ERK1/2 at Ser^298^. PIMT protein was fragmented into 2 parts, PIMT-N (1-334) and PIMT-C (330-853) and fused to GST. (**B**&**C**) GST-PIMT-N (**B**) and GST-PIMT-C (**C**) bound to glutathione sepharose beads were subjected to kinase reaction in the presence of HeLa nuclear extract [HNE] and constitutively active purified MAPKs ERK1 and ERK2. (**D**) Glutathione sepharose beads bound GST-PIMT-N [W] and GST-PIMT^S298A^ [M] were subjected to kinase assay with active and purified ERK2. Mutation at MAPK recognition site [PSSP] abolished phosphorylation of PIMT.(**E**)293T cells were transfected with vector or RAF-BXB and 24 h post transfection cells were treated with DMSO or 10 µM UO126 for 30 min. Subsequently cells were lysed and resolved on 10% SDS-PAGE and probed with Anti-pERK1/2. Blots were stripped and reprobed withAnti-ERK1/2. (**F** & **G**) 293T cells were transfected with pCMV-PIMT Flag (F, G) or pCMV- PIMT^S298A^ Flag (G) along with RAF-BXB (F, G) and cells were treated with UO126 where indicated (F). Post transfection cells were cultured in DMEM containing 1% FBS overnight, PIMT was immunoprecipitated with Anti-PIMT followed by separation on 10% SDS-PAGE and probed with Anti-MPM2 (F, G). Blots were stripped and reprobed with Anti-PIMT (F) or Anti-Flag (G) as mentioned.

To test this prediction, we carried out *in vitro* kinase assays with two overlapping purified GST fused fragments of PIMT: PIMT-N (1-334) and PIMT-C (330-853; Figure 1A). *In vitro* kinase assays with ERK1 and ERK2 revealed that PIMT-N was robustly phosphorylated by ERK2 but was minimally modified by ERK1 (Figure 1B). Both ERK1 and ERK2 failed to phosphorylate PIMT-C suggesting that PIMT contains only (one) ERK2 site (Figure 1C). Both GST fused protein fragments were phosphorylated by HeLa nuclear extract (HNE) which served as a positive control for the kinase reaction. No phosphorylation was noted with GST alone (data not shown). Further, purified recombinant ERK2 phosphorylated wild-type PIMT-N but failed to phosphorylate its point mutant (Ser^298^Ala; Figure 1D, lane M), confirming the authenticity of ERK2 phosphorylation site.

We next examined whether PIMT is phosphorylated at Ser^298^ in intact cells. For this, we overexpressed Flag tagged PIMT (WT) in the presence or absence of RAF-BXB in 293T cells. Post transfection, PIMT was immunoprecipitated using Anti-PIMT and was probed with ERK substrate antibody (Anti-MPM2) followed by re-probing with Anti-PIMT antibody. As shown in Figure 1F, overexpression of RAF-BXB enhanced the phosphorylation of PIMT. Inhibition of ERK pathway by UO126 blocked both RAF-BXB dependent ERK and PIMT phosphorylation (Figure 1E and 1F). Further, we observed that mutation of the ERK2 target site (Ser^298^Ala) abolished RAF-BXB dependent phosphorylation of PIMT (Figure 1G). Taken together, our results demonstrate that PIMT is a genuine substrate of ERK2. 

### Regulation of PIMT transcriptional potential *via* MAPK/ERK pathway

**Figure 2:**
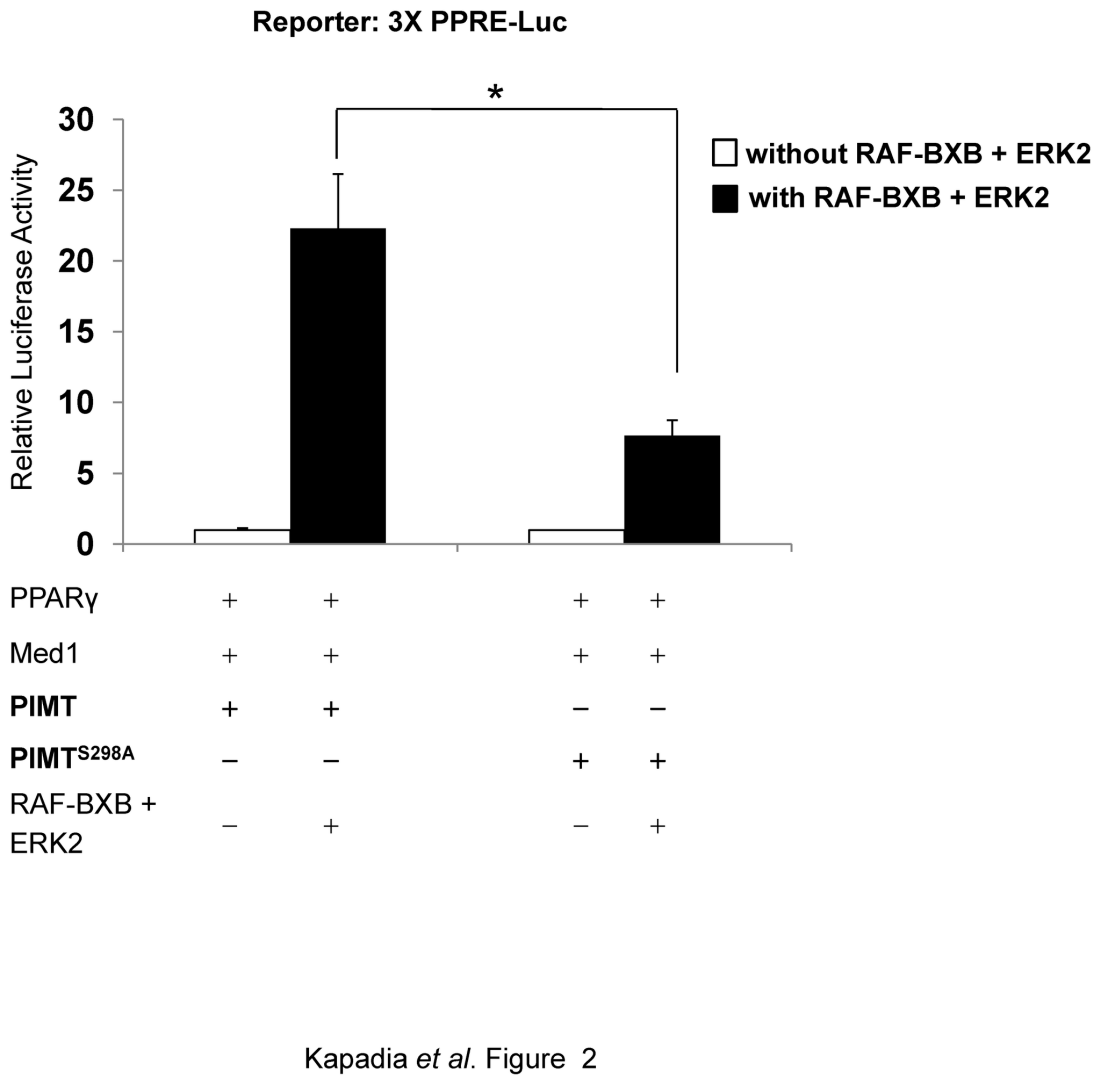
Phosphorylation of PIMT by RAF-BXB/ERK2 potentiates Med1 dependent transcriptional activity. HeLa cells were transiently cotransfected with 3X-PPRE driven luciferase construct and PPARγ, Med1 and PIMT or PIMT^S298A^ encoding constructs along with or without RAF-BXB and ERK2. Thirty hours post transfection, cells were lysed and luciferase activity was measured. The values were normalized with corresponding β-galactosidase activity and expressed relative to PIMT (column 1) which was set to 1. Data are representative of 5 independent experiments. Statistical analysis was performed using Student’s *t*-test (unpaired, two-tailed).* *p*<0.05.

Because MAPK/ERK signal transduction cascade was involved in the PIMT phosphorylation, it was necessary to elucidate the functional relevance of this phosphorylation. To demonstrate the effect of ERK-mediated PIMT phosphorylation, we transiently overexpressed MAPK inducers along with PPARγ and Med1 expression plasmids and 3XPPRE-Luc reporter construct in HeLa cells. We identified that RAF-BXB/ERK2 mediated phosphorylation of PIMT exerted an inductive effect (~21-fold) on Med1 mediated PPARγ driven transcriptional activity as assessed by luciferase activity (Figure 2). This inductive effect was significantly reduced (~ 2.4 fold, *p* <0.05) in the presence of PIMT^S298A^ (Figure 2) revealing the significance of ERK signal transduction pathway in PIMT mediated transcriptional regulation. This observation establishes the involvement of ERK2 in the regulation of PIMT activity and its potential role in modulating gene transcription.

### PIMT is recruited to PEPCK promoter

**Figure 3:**
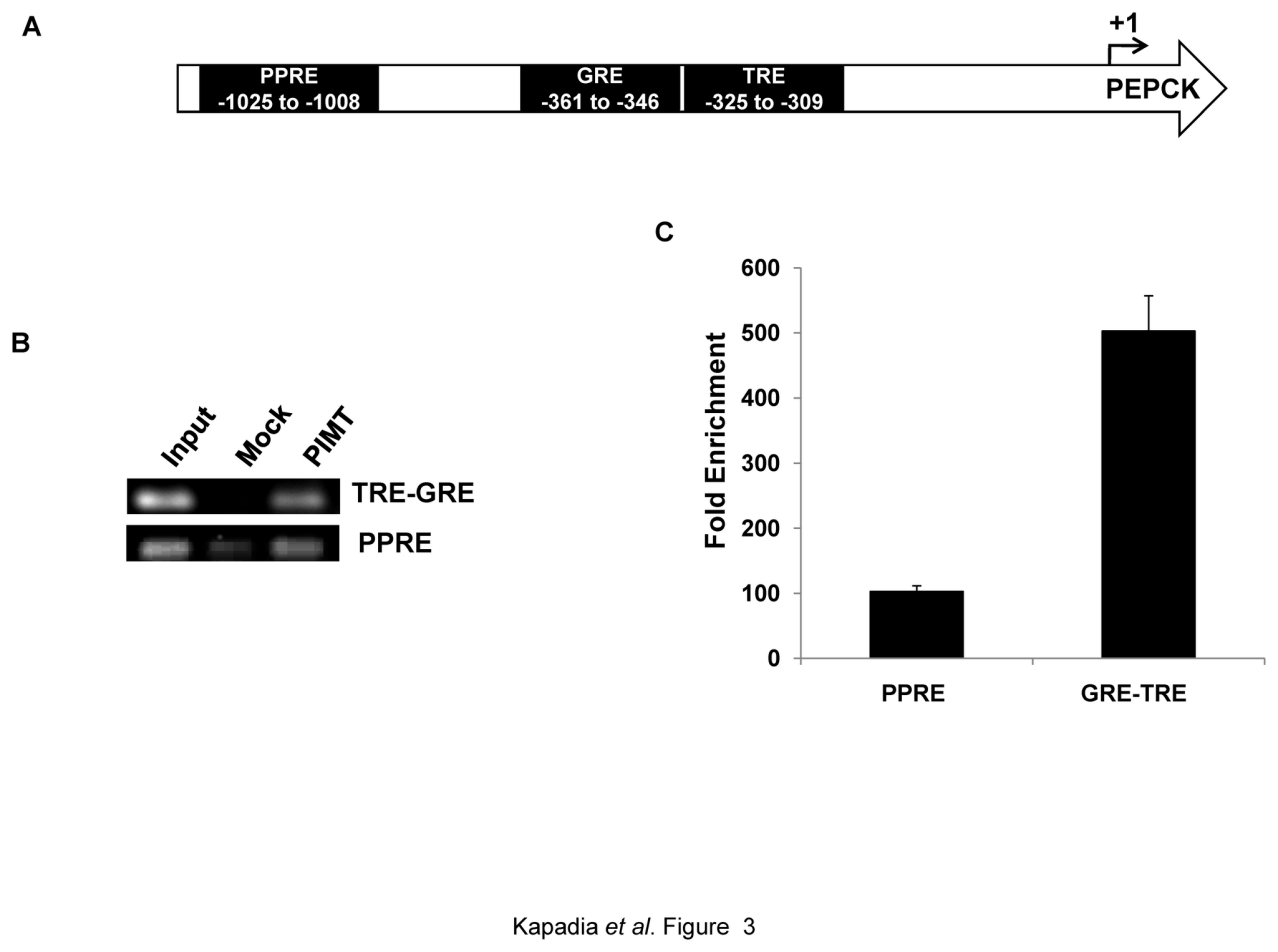
PIMT is recruited to PEPCK promoter. (**A**) A schematic diagram of PEPCK promoter showing PPRE, GRE and TRE. (**B**&**C**) Chromatin-immunoprecipitation assay was performed in HepG2 using Anti-PIMT or non specific Anti-Rabbit IgG on human PEPCK promoter (**B**)and quantified using ChIP qPCR(**C**).

We previously reported that PIMT enhances the transcriptional activity of PPARγ and RXRα utilizing 3XPPRE-luc reporter assays [1,6]. It is well established that Med1 plays a key role in facilitating ligand-dependent interactions of the coregulators with nuclear receptors [32]. Several studies have independently established that PEPCK transcription is under fine regulation via nuclear hormone receptors through response elements such as PPRE, GRE, IRE (HNF4α binding sites) and TRE located in the PEPCK promoter region (Figure 3A) [33,34]. In addition, many coregulators such as CBP/Ep300 [35] and PRIP/NCoA6 [36] modulate the expression of PEPCK, either directly or indirectly. Since PIMT has been reported to modulate transcriptional activity by influencing coactivator function and the expression of PEPCK is also tightly regulated by multiple coactivators[37,38], we examined whether PIMT plays a role in regulating PEPCK expression. To test this hypothesis we first tested whether PIMT is recruited to PEPCK promoter. Using chromatin immunoprecipitation (ChIP) assays followed by qPCR we observed that PIMT was indeed recruited to the PEPCK promoter (Figure 3B) under basal conditions and PIMT was recruited to TRE-GRE region more efficiently as compared to the PPRE region (Figure 3B and 3C). Thus, we conclude that PIMT is required for the basal expression of the PEPCK. 

### MAPK/ERK2-mediated phosphorylation of PIMT enhances PEPCK promoter activity

**Figure 4:**
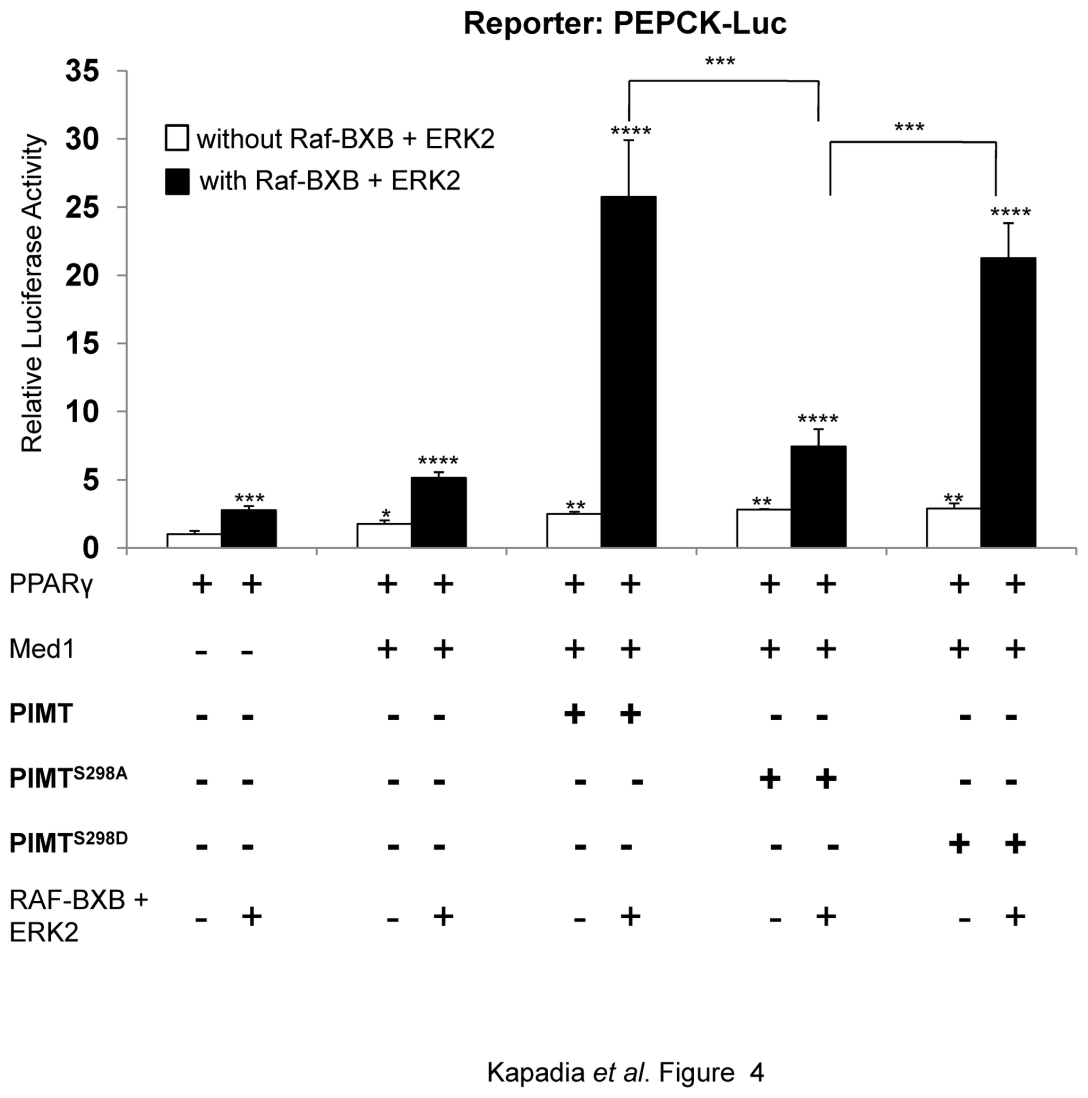
ERK2 dependent phosphorylation of PIMT increases PEPCK promoter activity. 293T were transfected with pGL3-PEPCK promoter and other constructs as mentioned in Figure 2 legend. Thirty hours post transfection, cells were lysed and luciferase activity was measured. The values were normalized with corresponding Renilla luciferase activity and expressed relative to PPARγ (unphosphorylated) (column 1) which was set to 1. Data are representative of 3 independent experiments. Statistical analysis was performed using one way ANOVA followed by Bonferroeni’s post hoc test. *p<0.001.

Above experiments demonstrated that ERK2 dependent phosphorylation of PIMT at Ser^298^ increases the transactivation ability of PPARγ and that PIMT is a part of PEPCK transcriptional apparatus. We hypothesized that ERK dependent phosphorylation of PIMT may also modulate the expression of PEPCK. To address this possibility, we used a PEPCK promoter-luciferase system. We transiently transfected 293T cells with the full length PEPCK promoter-luciferase gene along with other indicated constructs (Figure 4). In absence of MAPK inducers, Med1 enhanced PEPCK promoter activity approximately 2-fold while a ~4 fold increase was observed with PIMT-Med1 overexpression compared to PPARγ alone (Figure 4). Phosphorylation of Med1 and PIMT-Med1 by RAF-BXB and ERK2 significantly enhanced PEPCK promoter activity by ~5.0 and ~24.0 fold, respectively (Figure 4) suggesting that RAF-BXB/ERK2 cascade exerts a positive effect on Med1 and PIMT coactivator function. However, when PIMT^S298A^was overexpressed along with wild-type Med1 a substantial decrease (~3-fold) in transcriptional activity was observed. 

Mutation of serine to aspartate creates a negative charge in the residue that in part mimics the effects of the phosphorylated serine. Thus, we mutated Ser^298^ to Asp (a phosphomimetic mutation) and studied its effect on PIMT mediated transcriptional activation activity using promoter-reporter assays. Ser^298^Ala mutation (PIMT^S298A^) significantly reduced the promoter activity while the phosphomimetic mutation of PIMT (PIMT^S298D^) induced reporter activity to the levels comparable to that of WT PIMT observed in the presence of MAPK inducers. However, in the absence of RAF-BXB there was no change in the reporter activity upon overexpression of either PIMT or its mutants. Based on the above observations, it is reasonable to conclude that phosphorylated PIMT enhances its ability to promote PEPCK promoter activity and therefore PIMT may potentially play an important regulatory role in gluconeogenesis. 

### Overexpression of PIMT in primary rat hepatocytes increases hepatic glucose output in MAPK/ERK dependent manner

To further characterize the physiological significance of MAPK/ERK dependent PIMT phosphorylation in the regulation of hepatic gluconeogenesis, using adenovirus vectors we overexpressed wild type or Ser^298^ mutants of PIMT in primary rat hepatocytes along with Med1. Ad/eGFP was used as the control. Infection of cells with Ad/PIMT or Ad/Med1 increased the glucose output compared to Ad/eGFP infected cells (see Methods S1 for experimental details). Interestingly, relative to wild type PIMT infected hepatocytes; the glucose output was reduced in Ad/PIMT^S298A^ but not in Ad/PIMT^S298D^ infected cells.

**Figure 5:**
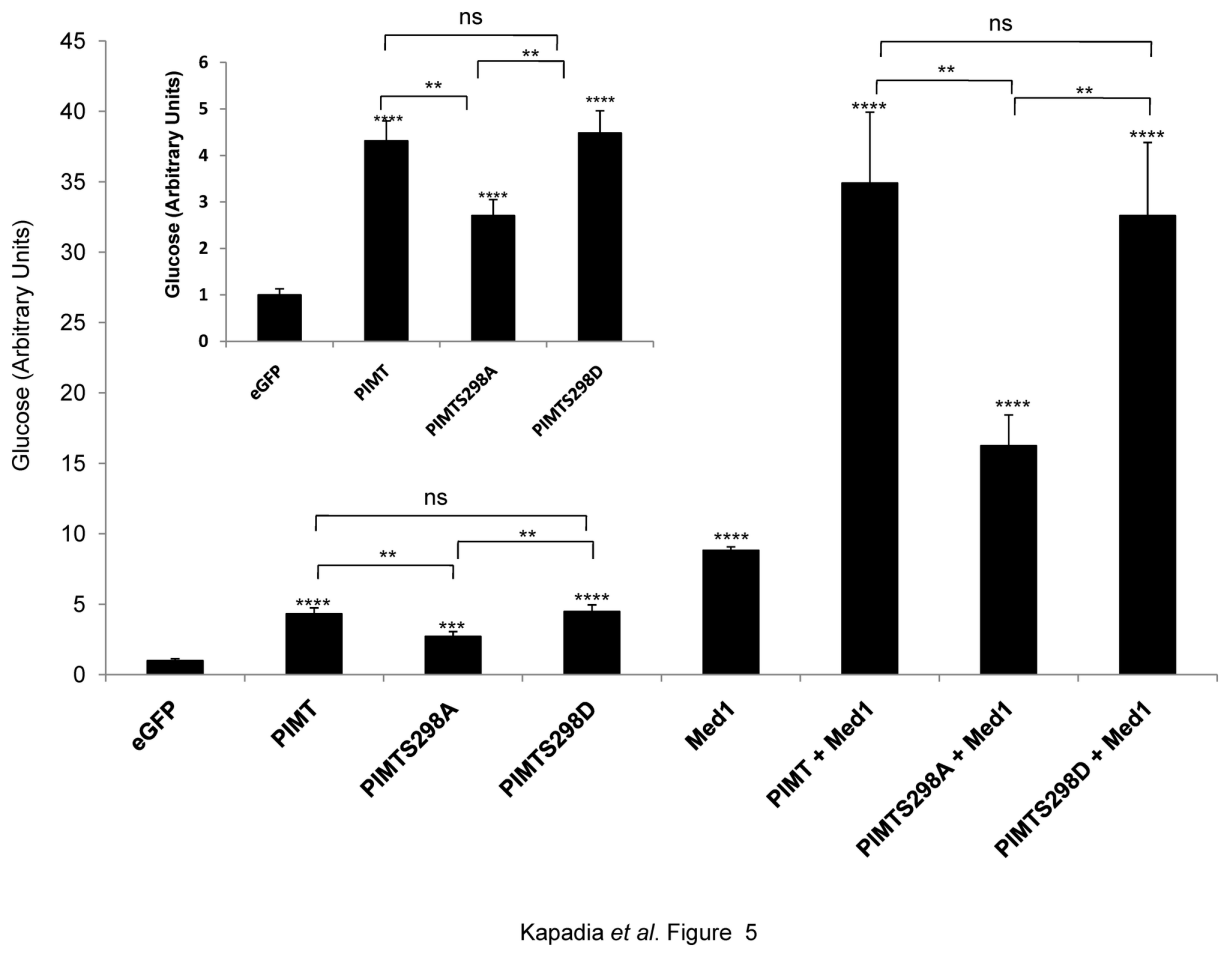
Phosphorylation of PIMT at Ser^298^ is essential for its ability to augment hepatic glucose output. Primary rat hepatocytes were infected with Ad/PIMT or Ad/PIMT^S298A^ or Ad/PIMT^S298D^ and/or Ad/Med1. Ad/eGFP infection was used as control. After 24 h of infection, the cells were cultured in glucose production medium for 6 h and amount of glucose released in cell supernatants was measured. The values were normalized to corresponding total protein content and expressed relative to Ad/eGFP control which was considered as 1. The inset shows the result of glucose output data in Ad/PIMT or Ad/PIMT^S298A^ or Ad/PIMT^S298D^ infected primary hepatocytes. Data are a representative of 3 independent experiments. Statistical analysis was performed using one way ANOVA followed Bonferroeni’s post-hoc test. ^**^ *p*<0.01, ^***^ *p*<0.005, ^****^ *p*<0.0001 *vs* Ad/eGFP.

Next, we assessed the effect of co-infection of PIMT or its mutants along with Med1 in primary rat hepatocytes and determined glucose output as above. An additive effect was observed upon overexpression of PIMT and Med1 compared to overexpression of PIMT or Med1 alone (Figure 5, Figure S2). Consistently, Ser^298^Asp, but not Ser^298^Ala mutant of PIMT showed additive effect on Med1 dependent glucose output (Figure 5). Thus, based on the promoter reporter gene assays and glucose output experiments we concluded that presence of negative charge on Ser^298^ of PIMT is indispensable in augmenting the hepatic glucose output.

We next investigated the functional implication of ERK pathway in PIMT-Med1 mediated hepatic glucose output. To address this question, we infected rat hepatocytes with Ad/PIMT and/or Ad/Med1 and cultured them in presence of UO126, a MEK1/ERK inhibitor. Upon infection with either Ad/PIMT or Ad/Med1 the glucose output was increased compare to that of Ad/LacZ infection, which was completely abolished in the presence of the MEK/ERK inhibitor (Figure S2). Also in co-infection of Ad/PIMT and Ad/Med1 in the presence of UO126, the glucose output was abrogated (Figure S2). Therefore, we conclude that PIMT and Med1 play a pivotal role in the control of hepatic glucose output in ERK dependent manner.

### Overexpression of PIMT in rat primary hepatocytes increases gluconeogenic gene expression in MAPK/ERK dependent manner

**Figure 6:**
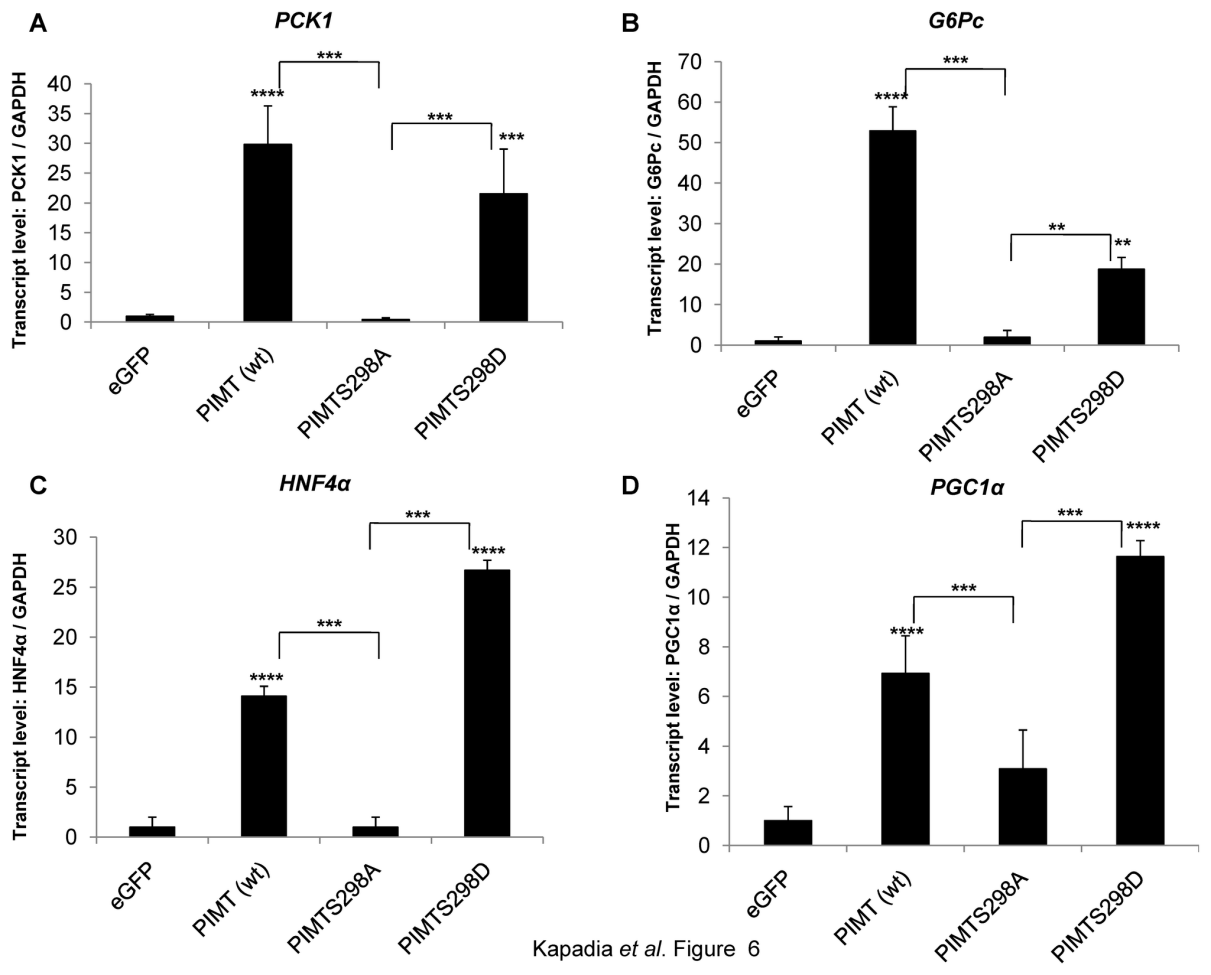
Phosphorylation of PIMT at Ser^298^ is required for its ability to enhance gluconeogenic genes. Primary rat hepatocytes were infected with Ad/eGFP (control) or Ad/PIMT or Ad/PIMT^S298A^ or Ad/PIMT^S298D^ and/or Ad/Med1. After 24 h of infection, total RNA was isolated and the expression of (**A**) PCK1, (**B**)*G6Pc*(**C**)HNF4α and (**D**)*PGC1α* was determined using qPCR. The values were normalized to corresponding GAPDH expression and expressed relative to Ad/eGFP control which was considered as 1. Data are a representative of 3 independent experiments. Statistical analysis was performed using one way ANOVA followed Bonferroeni’s post-hoc test. ^**^ *p*<0.01, ^***^ *p*<0.005, ^****^ *p*<0.0001 *vs* Ad/eGFP.

The above data showed that overexpression of PIMT enhanced hepatic glucose output in MAPK/ERK dependent manner. This prompted us to analyze the expression of gluconeogenic genes. Upon overexpression of PIMT, the mRNA levels of PEPCK (*PCK1*) and G6Pase (*G6Pc*), the rate limiting enzymes of gluconeogenesis, were observed to increase compared to that of Ad/eGFP supporting our observation that PIMT enhances hepatic glucose output (Figure 6). Consistent with reporter experiments, overexpression of PIMT^S298D^ but not PIMT^S298A^ augmented the expression of PEPCK and G6Pase genes in primary hepatocytes. Similar trend was also noted with the expression of HNF4α (transcription factor regulating gluconeogenic genes) and PGC1α (the master regulator of metabolism) (Figure 6). Further, consistent with hepatic glucose output experiments, the expression of the gluconeogenic genes was abolished in MEK/ERK inhibitor treated Ad/PIMT infected cells, (Figure S3). Taken together, our results demonstrate that phosphorylation of PIMT at Ser^298^ enhances gluconeogenesis via upregulation of the expression of gluconeogenic genes.

### Overexpression of PIMT in liver increases PEPCK mRNA level in mice

**Figure 7:**
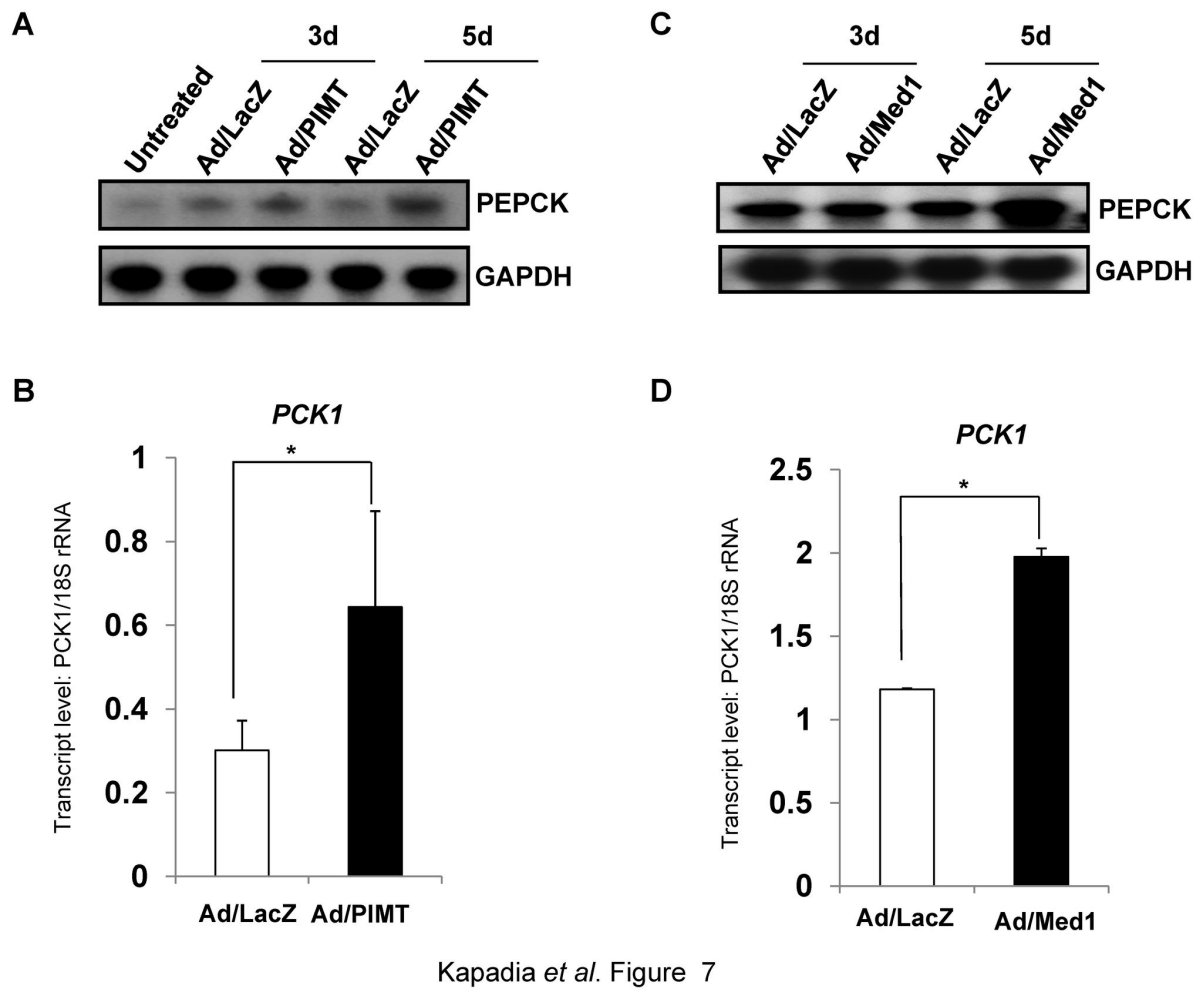
Overexpression of PIMT or Med1 increases hepatic PEPCK expression in mice. Mice were injected via tail vein with Ad/LacZ or Ad/PIMT (**A** & **B**) or Ad/Med1 (**C** & **D**). After 5 days of injection mice were sacrificed and total RNA was isolated from liver. The expression of PEPCK was determined by northern blotting (A and C) or by qPCR (**B** & **D**). Blots were re-probed for GAPDH to ensure equal loading (**A** & **C**).

Because PIMT was able to regulate PEPCK expression in isolated hepatocytes, we next examined whether PIMT would stimulate PEPCK in vivo. To test this, mice were injected with Ad/PIMT or Ad/Med1 and were sacrificed after 3^rd^ day or 5^th^ day of injection. Consistent with the above observation, upon overexpression of PIMT, PEPCK mRNA levels were increased post 5^th^ day of injection when compared to that of Ad/LacZ (Figure 7A and 7B). Similarly, PEPCK levels were also enhanced upon Med1 overexpression (Figure 7C and 7D). Overexpression of PIMT and Med1 in mice liver was confirmed in parallel by qPCR (Figure S4). 

### PIMT expression is required for gluconeogenesis in liver

**Figure 8:**
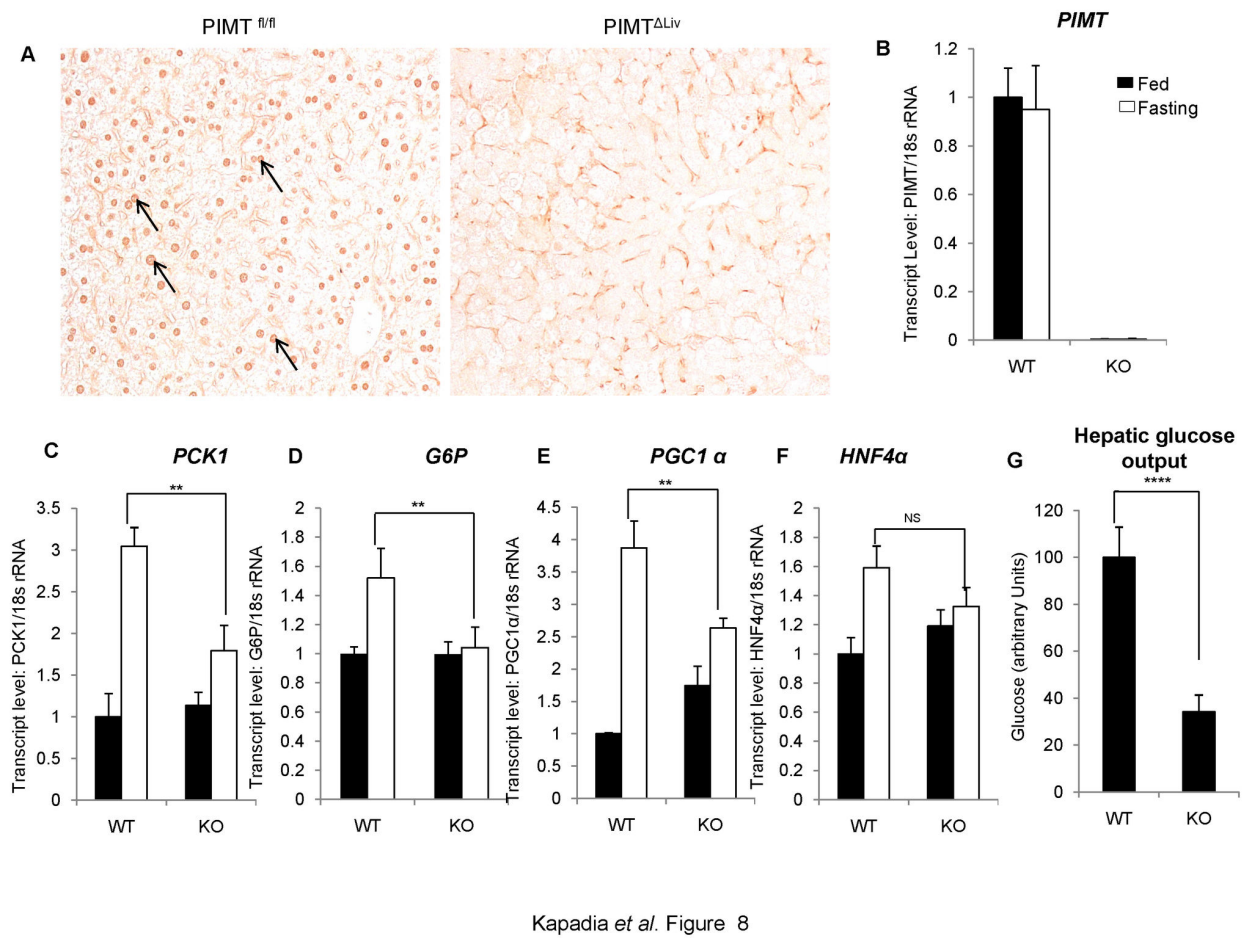
Ablation of PIMT expression in liver reduces gluconeogenesis (**A**) De-paraffinized 5 μm thin sections were immunohistochemically stained using PIMT antibody in WT and KO mice of liver tissue. (**B**) Total RNA was isolated from liver tissue and qPCR analysis of PIMT in PIMT^fl/fl^ (WT) and PIMT^ΔLiv^ (KO) livers was performed. (**C**-**F**) WT and KO mice were fasted for 72h. RNA was extracted and qPCR was performed for PCK1 (C), *G6P* (D), *PGC1α* (E) and HNF4α (F). (**G**) Primary hepatocytes were isolated from WT and KO mice. Post 24h of isolation, cells were culture in glucose production medium for 6 h. Amount of glucose released was estimated and the values were normalized to the corresponding protein content. The values are expressed relative to the normalized WT control group. Data are representative of three independent experiments. Statistical analysis was performed using unpaired Student’s *t*-test **p<0.01, ****p<0.001, NS: Non-Significant.

To firmly establish the role of PIMT in gluconeogenesis in liver and to complement the PIMT overexpression results described above, we have used mice in which PIMT gene was selectively deleted. We recently showed that PIMT is essential for normal development and that disruption of PIMT results in embryonic lethality at day 3.5 [4]. To study the liver specific functions of PIMT, using Cre-LoxP strategy, we recently created a mouse strain in which PIMT was selectively deleted in liver (PIMT^ΔLiv^KO mice)(Figure 8A and 8B) . These mice appear to grow normally but some of the liver specific metabolic pathways appear to be affected. The full description of these mice and the effects of PIMT deletion in various aspects of liver functions are currently under investigation and will be published elsewhere. For the studies to be described below, PIMT^fl/fl^ and PIMT^ΔLiv^KO were fasted for 72h, and total RNAs were prepared and the key enzymes in gluconeogenesis including PCK1, G6P, the coactivator PGC1α and the target of PGC1α, HNF4α, were quantified using qPCR assay. As shown in Figure 8C, during fasting, a striking increase in PCK1 and PGC1α levels (3 to 4-fold) were observed in PIMT^fl/fl^ mice. The enzyme G6P and HNF4α levels were also increased somewhat (1.5-fold). Importantly, in PIMT^ΔLiv^KO mice, these genes were not increased at all (see G6P and HNF4α) or increased only to modest levels (PCK1 and PGC1α). 

To determine whether the gene changes involved in gluconeogenesis reflect in glucose release from liver, we prepared hepatocytes from PIMT^fl/fl^ and PIMT^ΔLiv^KO mice, incubated with medium containing sodium lactate and sodium pyruvate and measured the release of glucose into medium after 6 h. In agreement with the *in vivo* data described above, glucose release was reduced significantly in hepatocytes derived from PIMT^ΔLiv^KO as compared to PIMT^fl/fl^ livers (Figure 8G). These data combined with the RNA levels related to PCK1 and PGC1α of PIMT^ΔLiv^KO livers (shown above) and our overexpression data presented in earlier sections of this manuscript unambiguously show that PIMT plays a critical role in gluconeogenesis.

### Thyroid hormone stimulates ERK-mediated PIMT phosphorylation and enhances hepatic gluconeogenesis in rats

**Figure 9:**
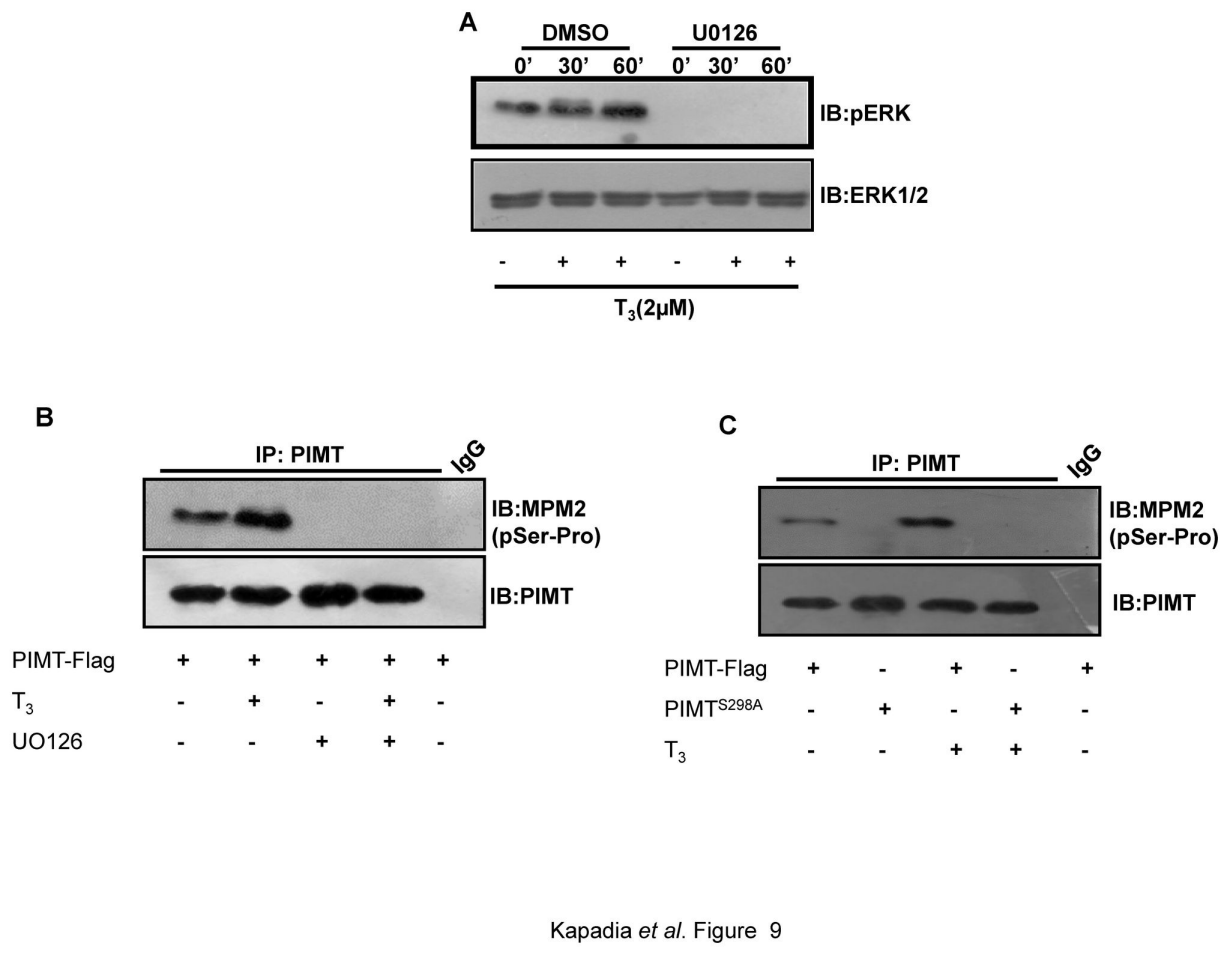
Thyroid hormone induced ERK activation is required for phosphorylation of PIMT at Ser^298^. (**A**) 293T cells were stimulated with T_3_ (2µM) or vehicle control DMSO for the indicated time points and the phosphorylation of pERK1/2 was examined by western blotting. Blots were re-probed with ERK1/2 antibody to ensure equal loading. (**B**-**C**) 293T cells were transfected with pCMV-PIMT Flag (B, C) or pCMV- PIMT^S298A^ Flag (C) and cells were treated with or without T_3_ (2µM) (B,C) and UO126 where indicated (B). Post transfection cells were cultured in DMEM containing 1% FBS overnight followed by separation on 10% SDS-PAGE and probed with Anti-MPM2 (B, C). Blots were stripped and reprobed with Anti-PIMT (B, C).

**Figure 10:**
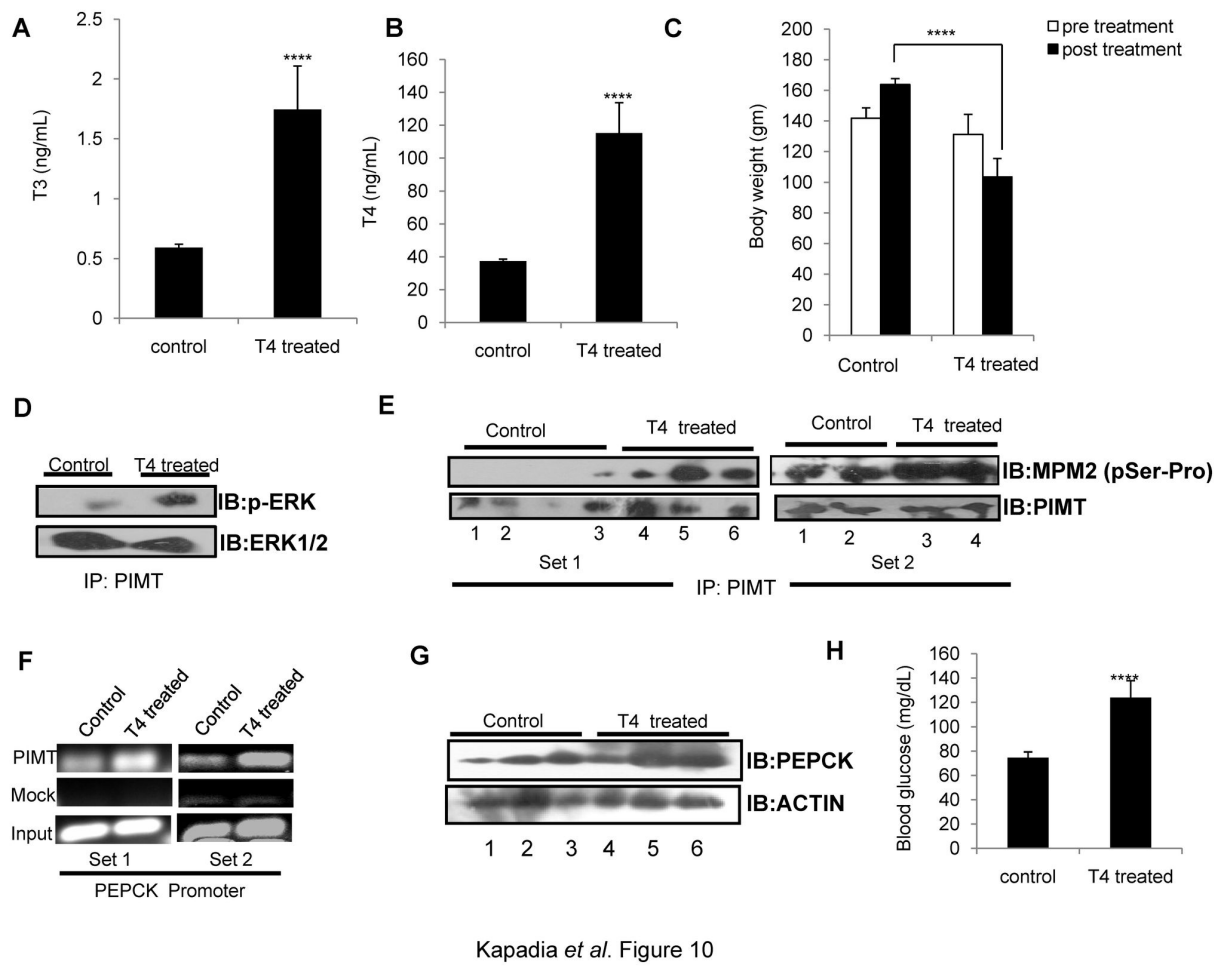
Thyroid hormone mediated ERK activation stimulates PIMT phosphorylation and enhances hepatic gluconeogenesis in rats. Rats (n=5) were injected intraperitoneally (0.1mL/animal) with L-thyroxine solution for 2 weeks. Post treatment animals were sacrificed, serum was collected for detecting levels of T_3_ (**A**) and T_4_ (**B**) and whole body weight (**C**) were measured. (**D**) PIMT immunoprecipitated lysates from liver tissue were analyzed by western blotting with phospho-ERK1/2 and total ERK1/2 antibodies. (**E**)PIMT was immunoprecipitated from liver tissue lysates (Set1: 3 control and 3 treated; Set2: 2 control and 2 treated) separated on 10% SDS PAGE and probed with Anti-MPM2 followed by Anti-PIMT as mentioned. (**F**) Chromatin immunoprecipitation assay was performed in liver tissue lysates (one control and one treated in both Set1 and Set2) using Anti-PIMT or mock Anti-goat IgG on rat PEPCK promoter. (**G**) The liver homogenates were analyzed using Anti-PEPCK (top panel) or Anti beta actin (bottom panel). (**H**) Overnight fasting blood sugar was measured using glucose strip.

We noted that ERK mediated phosphorylation of PIMT enhances hepatic gluconeogenesis. Thyroid hormone has been reported to increase MAPK/ERK phosphorylation [18,39,40]. Also, the recruitment of PIMT to PEPCK promoter was more pronounced in TRE-GRE region. This prompted us to study the role of ERK-mediated phosphorylation of PIMT in thyroid hormone regulated hepatic gluconeogenesis. For this, we examined the effect of thyroid hormone on the phosphorylation status of PIMT as described above (Figure 1). Here, we found that the stimulation of 293T cells with T_3_ enhanced the levels of pERK1/2 (Figure 9A) and the phosphorylation of PIMT at the ERK2-target site Ser^298^ (Figure 9C) in an ERK-dependent manner (Figure 9B). Furthermore, systemic administration of T_4_ to rats resulted in significant increase of the plasma levels of T_4_ and T_3_ (Figure 10A and 10B) and decrease in the body weight (Figure 10C) of the treated animals compared to controls demonstrating that animals were hyperthyroid. Thyroid hormone administration enhanced levels of pERK (Figure 10D) and increased ERK-dependent phosphorylation of PIMT at Ser^298^ (Figure 10E) in the livers of the hyperthyroid animals. Also, ChIP assays showed that the recruitment of PIMT was increased on the PEPCK promoter in liver of hyperthyroid rats (Figure 10F). In addition, the expression of PEPCK and serum glucose levels were also found to be elevated (Figure 10G and 10H).Taken together, these results establish that PIMT is phosphorylated in intact liver cells and that this pathway is functional in the liver at the physiological levels of PIMT and PEPCK and PIMT and augments hepatic gluconeogenesis.

## Discussion

PIMT/ NCoA6IP is an important component of the nuclear receptor signaling cascade which may act as a molecular bridge between chromatin remodeling and mediator complexes for modulating transcriptional activity [6]. The yeast homolog of PIMT, designated Trimethylguanosine synthase 1 (TGS1), has been shown to exhibit trimethylguanosine synthase activity, the methyltransferase responsible for the formation of 2, 2, 7-trimethylguanosine (m3G) 5’-cap structure of snRNAs and snoRNAs[2,41] while the *Drosophila* homolog designated as DTL was reported to be important for the development [3]. Therefore, PIMT appears to exhibit dual cellular functions in that it serves as a transcription cofactor [1,6,42], and also as an RNA hypermethylating enzyme [43]. Post-translational modifications such as phosphorylation play a crucial role in determining the contextual functionality of coactivators and other important components of the transcriptional apparatus in the regulation of gene expression [44].

Previous work from our and other laboratories strongly implicated kinase-dependent phosphorylation events in the regulation of recruitment and activity of transcription factors and co-activator proteins [17,18,45,46]. For example, Med1, an important subunit of the mediator complex, is a substrate of ERK2/5 and that the co-activator potential of Med1 is enhanced by phosphorylation [17]. Phosphorylation of CREB by PKA is essential for its transcriptional activation [45]. Further, TORC2, the co-activator of CREB, is sensitive to modification by the energy sensor AMPK and phosphorylation of TORC2 diminished its transcriptional potential by blocking its nuclear accumulation [46]. These and other examples highlight the significance of the cross talk between the kinase signaling and the regulation of gene expression programs. 

To delineate the effect of kinase signaling pathways in the regulation of PIMT function, we carried out phospho mapping of PIMT. *In silico* analysis identified a potential MAPK/ERK target site on the N-terminal of PIMT protein. Using *in vitro* kinase assays and cellular phosphorylation experiments, we established that PIMT contains one ERK2 target site (Ser^298^) which is located in the context of ERK1/2 consensus sequence, PX**S/T**P (PS**S**P) [27]. Mutation of Ser^298^ of PIMT markedly reduced ERK2-dependent modification, substantiating the authenticity of this site. ERK is required for its nuclear receptors transcriptional coactivator activity, as determined by transient-transcription assays (Figure 2). We observed that overexpression of PIMT boosted Med1-mediated transcriptional activity of PPARγ suggesting that the phospho-modification of PIMT may be functionally relevant. 

PEPCK is a critical regulator of glucose homeostasis under nutritional depletion condition and/or hormonal changes. Numerous hepatic nuclear receptors including PPAR, TRβ1, HNF4α, FOXO1 and co-activators like PGC1α, CBP/Ep300, PRIP/NCoA6 and SRC1/2 are bound to PEPCK promoter either directly or indirectly and are essential for gluconeogenic response [20,47-51]. Since PIMT is recruited to PEPCK promoter under basal conditions, we assessed the contribution of PIMT in the regulation of gluconeogenesis. Indeed, overexpression of PIMT in presence of MAPK inducers augmented the PEPCK promoter activity which was consistent with our 3XPPRE-Luc data (Figure 2). To further assess the regulatory role of PIMT, we used adenoviral mediated overexpression system. We identified that overexpression of PIMT (wt) promotes PEPCK expression in both cultured cells and in the mouse liver. Interestingly, single mutation of PIMT (PIMT^S298A^) significantly reduced the expression of PEPCK. In contrast to phosphodeficient mutation (PIMT^S298A^), phosphomimetic mutation (PIMT^S298D^) of PIMT restored its ability to enhance PEPCK expression. Consistently, we noted that glucose output was also enhanced in PIMT or PIMT^S298D^ but not PIMT^S298A^ overexpressing primary hepatocytes. Further, we showed that PIMT and its interacting partner Med1, a key component of mediator complex, additively enhanced glucose production. Moreover, overexpression of PIMT^S298D^ but not PIMT^S298A^ along with Med1 enhanced hepatic glucose output. Taken together, our data suggest that phosphorylation of PIMT at Ser^298^ leads to enhanced hepatic gluconeogenesis via modulating the expression of PEPCK and other gluconeogenic genes. 

We also observed that the pro-gluconeogenic action of PIMT or Med1 requires the intact MEK1/ERK signaling. PIMT or Med1 dependent increase in hepatic glucose output was inhibited upon the pharmacological blockade of signaling through MEK1/ERK pathway. This was consistent with our current and previous findings that phosphorylation of PIMT and Med1 augmented their transcriptional activating potential [17]. Our study identifies a critical role for ERK pathway in the control of hepatic gluconeogenesis. However earlier studies showed that phosphatidyl inositol 3 kinase (PI3K)/Akt but not Ras/MEK/ERK pathway is essential for insulin dependent regulation of PEPCK transcription reiterating the fact that different signaling cascades differentially regulate gene transcriptional programs under varied contexts [52,53]. 

To unmask the impact of PIMT in context of hepatic gluconeogenesis, we used PIMT^ΔLiv^ KO mice and analyzed the consequences on the expression of the gluconeogenic genes upon PIMT deletion. The expression of PEPCK and G6P, the two rate limiting enzymes of hepatic gluconeogenesis and PGC1α, the master regulator of metabolism, were noted to be significantly decreased upon PIMT deletion. Also hepatocytes isolated from liver of PIMT^ΔLiv^ KO mice showed dwindled glucose production. Thus, our data strongly implicate the pivotal role of PIMT in regulating the hepatic gluconeogenesis.

Data obtained in this study are in striking contrast with the observations of Xu*et al* [54]. Overexpression of MAP kinase phosphatase 3 (MKP3), ERK1/2-specific phosphatase, modestly increased glucose output of rat hepatoma cells under basal or dexamethasone and cAMP stimulated conditions suggesting a negative regulatory role of ERK pathway in gluconeogenesis [54]. The observed discrepancy could be originating due to different cells and conditions used in the two studies. In this study, we used more physiological relevant freshly isolated rat primary hepatocytes in comparison to a hepatoma cell line used in other study [54]. 

To explain the role of ERK2-mediated phosphorylation in pro-gluconeogenic action of PIMT, we propose two potential mechanisms. The phosphorylation status may lead to a conformational change in PIMT to its active form leading to enhanced transcriptional activity or phosphorylation of PIMT may increase the protein-protein interaction with Med1. Both these possibilities are not mutually exclusive. From our reporter assay, gene expression analysis of gluconeogenesis and hepatic glucose output assay, it is reasonable to conclude that the presence of negative charge on Ser^298^ of PIMT augments its activity. However, the degree of association between PIMT and Med1 was observed to be independent of their phosphorylation status (Figure S5.) Another possibility is the enhanced recruitment of protein(s) into the multi-protein transcriptional initiation complex upon PIMT phosphorylation, which could potentially modify the chromatin accessibility and activity at the PEPCK promoter. For instance, recently it was shown that PI3K/Akt dependent phosphorylation of Med1 facilitated recruitment and interactions of FoxoA1, Pol II and TBP leading to chromatin looping of UBE2C oncogene [55]. Also ERK mediated phosphorylation of Med1 enhanced its interaction with other mediator complexes, but its nuclear receptor interaction remains unaltered [32]. Future experiments will be directed to examine these possibilities. 

Gluconeogenic program is also positively regulated by CREB which is activated during fasting through its coactivator TORC2 and CBP/Ep300 and via the transcriptional up-regulation of another coactivator protein PGC1α [46,49]. In our previous study, we established that PIMT interacts with Med1 and CBP/Ep300, but we observed no direct binding between PIMT and PGC1α [6], thus we speculate that PIMT and PGC1α may not be directly co-associated in the regulation of PEPCK transcription. Considering the presence of putative cAMP responsive elements in PIMT promoter region, it will be important to investigate whether CREB cooperates with PIMT (similar to PGC1α which is a cAMP responsive gene) in controlling gluconeogenic genes expression.

**Figure 11:**
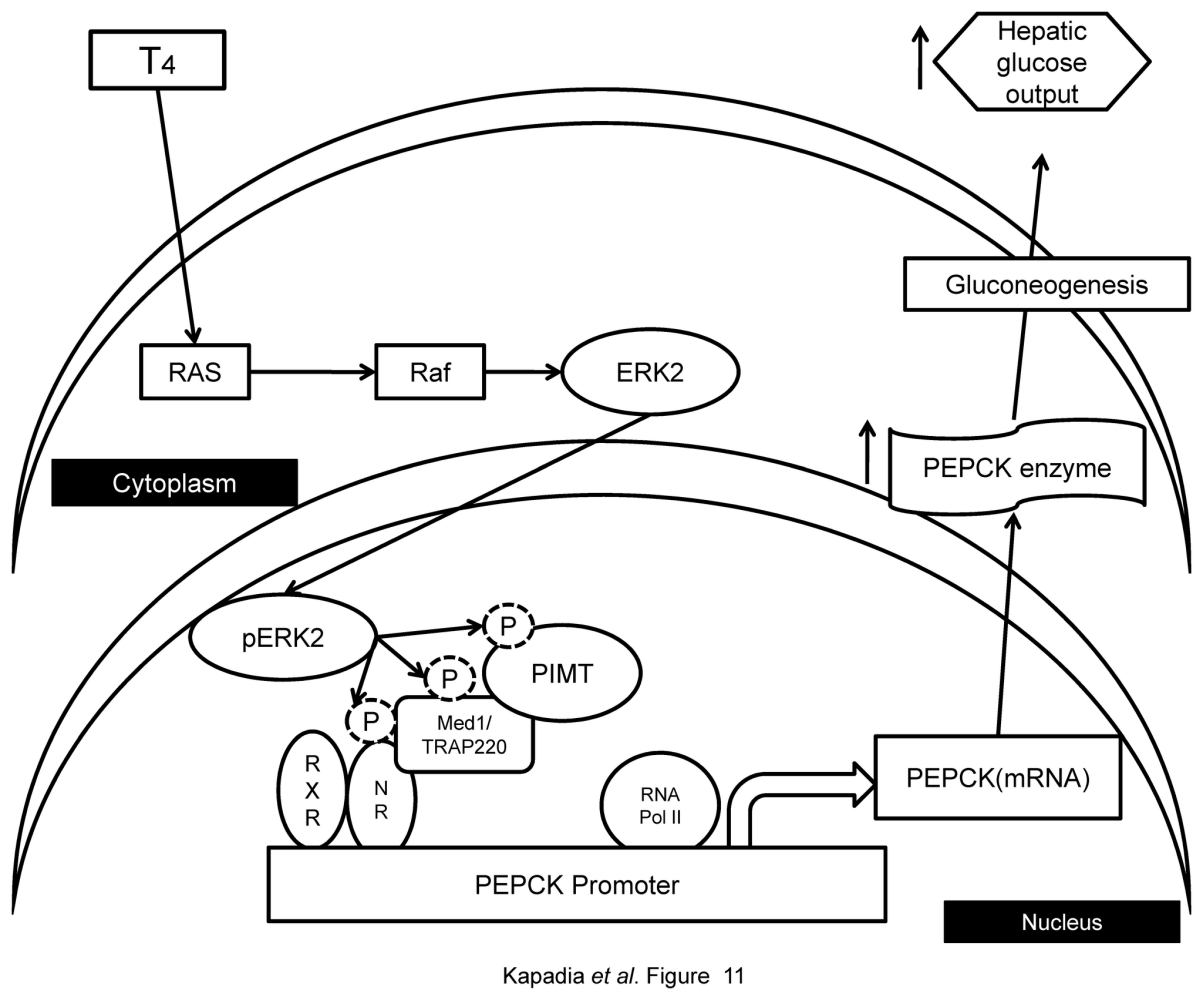
Schematicrepresentation of MAPK/ERK mediated PIMT-dependent regulation of hepatic gluconeogenesis. In response to MAPK induction by T_4_, ERK2 phosphorylates PIMT (at Ser^298^ position) which is in complex with Med1 and nuclear receptor (NR). Phosphorylation of PIMT increases Med1-dependent transcriptional activity of gluconeogenic genes leading to enhanced hepatic gluconeogenesis.

It should also be noted that several conditions such as thyroid stimulation [18,39,40], dyslipedemia, inflammation and hyperglycemia [56] augment MAPK/ERK activity. In particular, we have studied the effect of thyroid hormone on PIMT phosphorylation and its recruitment to PEPCK promoter .Activation of MAPK/ERK pathway under thyroid stimulation resulted in enhanced phosphorylation of PIMT at Ser^298^,which was associated with increased recruitment to PEPCK promoter leading to increased hepatic gluconeogenesis confirming that PIMT is phosphorylated in intact liver cells and that this pathway is functional in the liver at native levels of PIMT and PEPCK (Figure 11).

Previous studies have shown that TGS1, the homolog of human PIMT, hypermethylates the cap structure of snRNAs and snoRNAs in yeast [2]. A nuclear isoform of human PIMT was shown to possess RNA methyl transferase activity and was reported to promote TMG capping of some HIV RNAs [57]. Thus, it is tempting to determine the role of PIMT RNA methyl transferase activity, if any, in its pro-gluconeogenic action. Previous studies have focused on the RNA methyl transferase activity of PIMT [2,3,43,57] but to our knowledge this is the first study which identifies a functional role of PIMT in the transcriptional regulation of a native gene and thus our findings add a new dimension to the function of this protein in gene regulation. Considering the regulatory role of PIMT in hepatic gluconeogenesis, it is important to investigate the mechanisms that modulate the functions of PIMT. 

In summary, we demonstrated that the activity of PIMT is regulated by ERK2-dependent phosphorylation. Presence of negative charge at Ser^298^ seems to play a crucial role in increasing the PIMT mediated transcriptional activity. Collectively, the data obtained in this study strongly implicate PIMT as an important modulator of hepatic gluconeogenesis. Development of small molecules that could interfere with the phosphorylation or association of PIMT with Med1 might thus offer us novel therapeutic options to effectively treat Type 2 Diabetes.

## Supporting Information

Figure S1 **PIMT harbors consensus MAPK/ERK phosphoacceptor site and docking domains (**A**)** Schematic diagram of PIMT protein domain. PIMT contains RNA binding domain, S-adenosyl methionine binding domain overlapping with RNA Methyltransferase domain. The location of SP site (target site of MAPK/ERK) and the two putative conserved MAPK/ERK docking sites are also indicated. (**B**) Alignment of PIMT amino acid sequences across different mammalian species. SP site (MAPK/ERK target site) is conserved across the species. D-domain (hydrophobic amino acid rich domain) and DEF motif (XxxP, where X can be any aromatic amino acid and xx can be any hydrophobic amino acid) are also observed to be highly conserved.
(TIF)

Figure S2 **Overexpression of PIMT or Med1 enhances hepatic glucose output in MEK1/ERK dependent manner (A)** Primary rat hepatocytes were infected with control Ad/LacZ or Ad/PIMT eGFP and/or Ad/Med1. After 24 h of infection, the cells were cultured in glucose production medium for 6 h in presence of DMSO or UO126 (25μM) and amount of glucose released in cell supernatants were measured. The values were normalized to corresponding total protein content and expressed relative to Ad/LacZ (without inhibitor) control which was considered as 1. Data are representative of 3 independent experiments. Statistical analysis was performed using one way ANOVA followed by Bonferroeni’s post-hoc test.*p<0.001, # p<0.001 vs Ad/LacZ (column 1) and @p<0.0001vs Ad/PIMT eGFP and Ad/Med1 (DMSO treated, column 7). (**B**) DMSO or U0126 treated rat hepatocytes were lysed and analyzed by western blotting with phospho-ERK1/2 and total ERK1/2 antibodies. (**C**) Rat hepatocytes infected with Ad/PIMT eGFP were treated with DMSO or U0126 (25 µM) and the number of GFP positive cells (minimum 150 cells for each treatment) was counted using fluorescent microscope. (**D**) Rat hepatocytes were infected with Ad/LacZ or Ad/PIMT eGFP and expression of PIMT was analyzed by western blotting with anti-GFP antibody.
(TIF)

Figure S3 **Overexpression of PIMT enhanced expression of gluconeogenic genes in primary hepatocytes in MEK1/ERK dependent manner** Primary rat hepatocytes were infected with Ad/PIMT eGFP and Ad/Med1 or Ad/LacZ (control). Post 6 h of infection cells were cultured either in DMSO or treated with 25µM of UO126. After 24 h of infection, RNA was isolated and expression of (**A**) *PCK1*, (**B**) *G6Pc* (**C**) *HNF4α*, (**D**) *PGC1α*, (**E**) *PIMT* and (**F**) *Med1* was measured using qPCR. The values were normalized with the 18S rRNA and expressed relative to Ad/LacZ (DMSO) as 1. Statistical analysis was performed using one way ANOVA followed Bonferroni's post hoc test to determine the difference between the test result. *p<0.05, **p<0.005, ***p<0.001vs Ad/LacZ.
(TIF)

Figure S4 **Overexpression of PIMT and Med1 in mouse liver** qPCR analysis to confirm the over-expression of (**A**) *PIMT* and (**B**) *Med1* in the liver of wild type mice injected with Ad/PIMT eGFP and Ad/Med1 respectively.
(TIF)

Figure S5 **PIMT- Med1 interaction is independent of their phosphorylation state (**A**)** HeLa cells were transfected with Flag-tagged PIMT and Med1 along with or without Raf-BXB and ERK2. Nuclei were stained by DAPI (blue). FITC labeled anti FLAG and Cy3 labeled secondary antibody against Med1 were used to visualize the localization of PIMT and Med1, respectively using Deltavision deconvulation microscopy. (**B**) ^35^S labeled *in* *vitro* translated PIMT-N was and incubated for 2 h at 4°C with GST-Med1-C either unmodified or phosphorylated by purified ERK2. Following GST pull-down samples were resolved by SDS-PAGE and signals were visualized by autoradiography. (**C**) *In* *vitro* translated ^35^S labeled full length Med1 (Med1-FL) was incubated for 2 h at 4°C with GST-PIMT-N either unmodified or phosphorylated by purified ERK2. After incubation, PIMT-N was precipitated using glutathione sepharose beads followed by SDS-PAGE and autoradiography.
(TIF)

Methods S1 **Supporting Materials and Methods.**
(DOCX)

Table S1 **Primer sequences.** Sequences of the primers used in this study are provided. Nucleotides in bold represent mutations and primers prefixed with letter “Q” were used for qPCR analysis.
(DOCX)
