# Supplementary figures and images for: ERK2-Mediated Phosphorylation of Transcriptional Coactivator Binding Protein PIMT/NCoA6IP at Ser298 Augments Hepatic Gluconeogenesis
Source: PLoS One. 2013 Dec 17;8(12):e83787. doi: 10.1371/journal.pone.0083787 (PMC3866170; doi:10.1371/journal.pone.0083787)

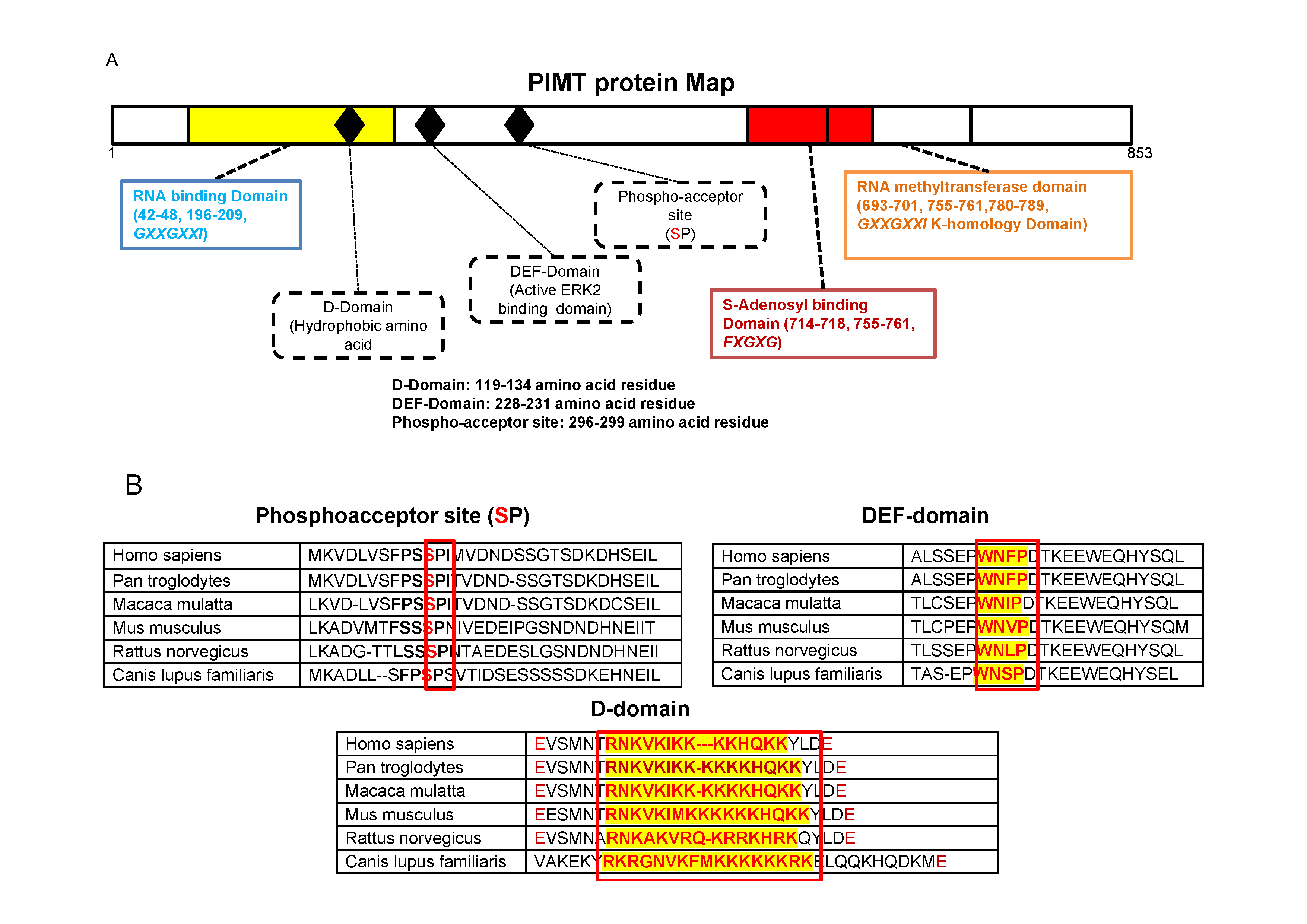

Supplement: Figure S1 — PIMT harbors consensus MAPK/ERK phosphoacceptor site and docking domains (A) Schematic diagram of PIMT protein domain. PIMT contains RNA binding domain, S-adenosyl methionine binding domain overlapping with RNA Methyltransferase domain. The location of SP site (target site of MAPK/ERK) and the two putative conserved MAPK/ERK docking sites are also indicated. (B) Alignment of PIMT amino acid sequences across different mammalian species. SP site (MAPK/ERK target site) is conserved across the species. D-domain (hydrophobic amino acid rich domain) and DEF motif (XxxP, where X can be any aromatic amino acid and xx can be any hydrophobic amino acid) are also observed to be highly conserved. (TIF) [file pone.0083787.s001.tif]

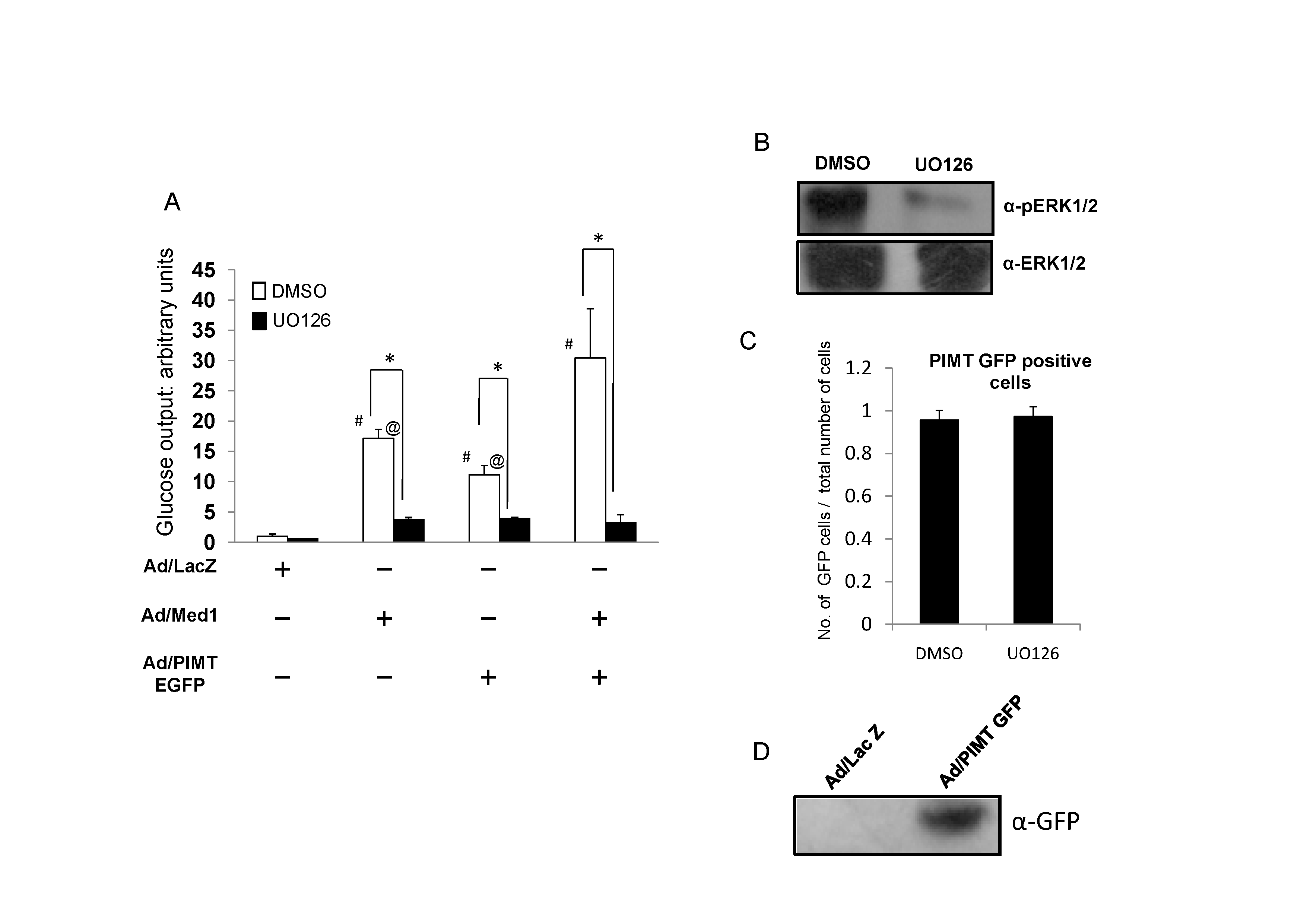

Supplement: Figure S2 — Overexpression of PIMT or Med1 enhances hepatic glucose output in MEK1/ERK dependent manner (A) Primary rat hepatocytes were infected with control Ad/LacZ or Ad/PIMT eGFP and/or Ad/Med1. After 24 h of infection, the cells were cultured in glucose production medium for 6 h in presence of DMSO or UO126 (25μM) and amount of glucose released in cell supernatants were measured. The values were normalized to corresponding total protein content and expressed relative to Ad/LacZ (without inhibitor) control which was considered as 1. Data are representative of 3 independent experiments. Statistical analysis was performed using one way ANOVA followed by Bonferroeni’s post-hoc test.*p<0.001, # p<0.001 vs Ad/LacZ (column 1) and @p<0.0001vs Ad/PIMT eGFP and Ad/Med1 (DMSO treated, column 7). (B) DMSO or U0126 treated rat hepatocytes were lysed and analyzed by western blotting with phospho-ERK1/2 and total ERK1/2 antibodies. (C) Rat hepatocytes infected with Ad/PIMT eGFP were treated with DMSO or U0126 (25 µM) and the number of GFP positive cells (minimum 150 cells for each treatment) was counted using fluorescent microscope. (D) Rat hepatocytes were infected with Ad/LacZ or Ad/PIMT eGFP and expression of PIMT was analyzed by western blotting with anti-GFP antibody. (TIF) [file pone.0083787.s002.tif]

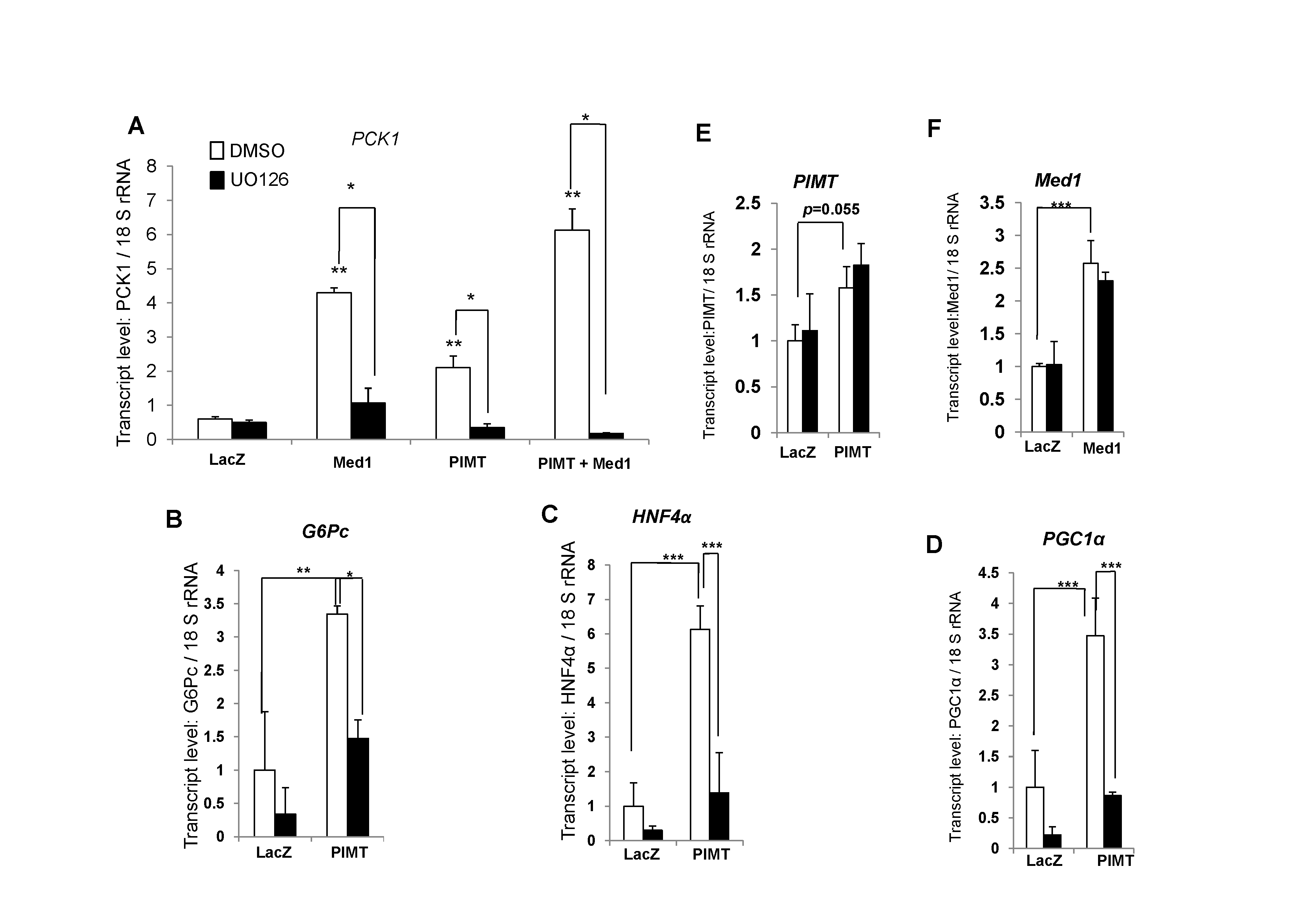

Supplement: Figure S3 — Overexpression of PIMT enhanced expression of gluconeogenic genes in primary hepatocytes in MEK1/ERK dependent manner Primary rat hepatocytes were infected with Ad/PIMT eGFP and Ad/Med1 or Ad/LacZ (control). Post 6 h of infection cells were cultured either in DMSO or treated with 25µM of UO126. After 24 h of infection, RNA was isolated and expression of (A) PCK1, (B) G6Pc (C) HNF4α, (D) PGC1α, (E) PIMT and (F) Med1 was measured using qPCR. The values were normalized with the 18S rRNA and expressed relative to Ad/LacZ (DMSO) as 1. Statistical analysis was performed using one way ANOVA followed Bonferroni's post hoc test to determine the difference between the test result. *p<0.05, **p<0.005, ***p<0.001vs Ad/LacZ. (TIF) [file pone.0083787.s003.tif]

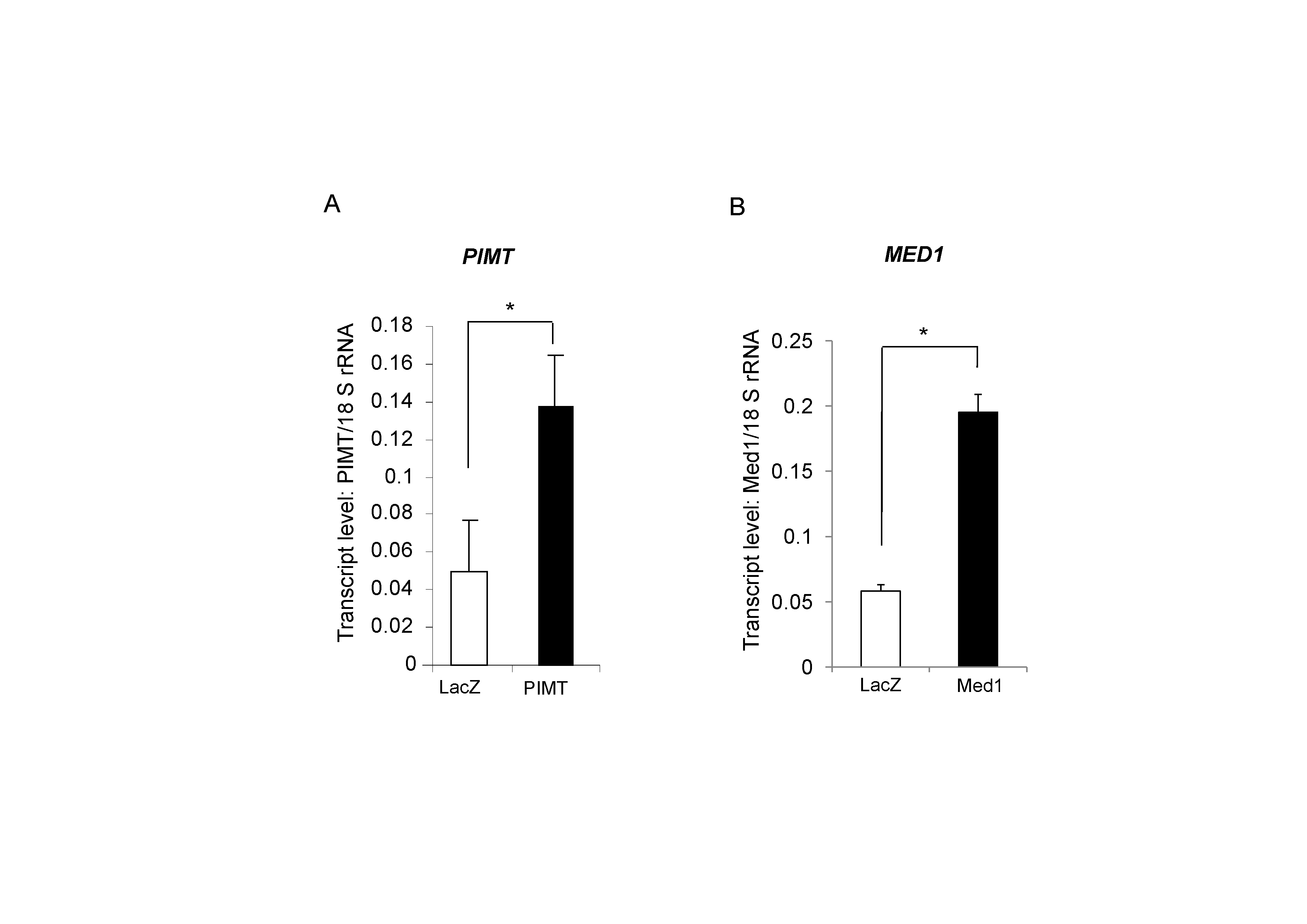

Supplement: Figure S4 — Overexpression of PIMT and Med1 in mouse liver qPCR analysis to confirm the over-expression of (A) PIMT and (B) Med1 in the liver of wild type mice injected with Ad/PIMT eGFP and Ad/Med1 respectively. (TIF) [file pone.0083787.s004.tif]

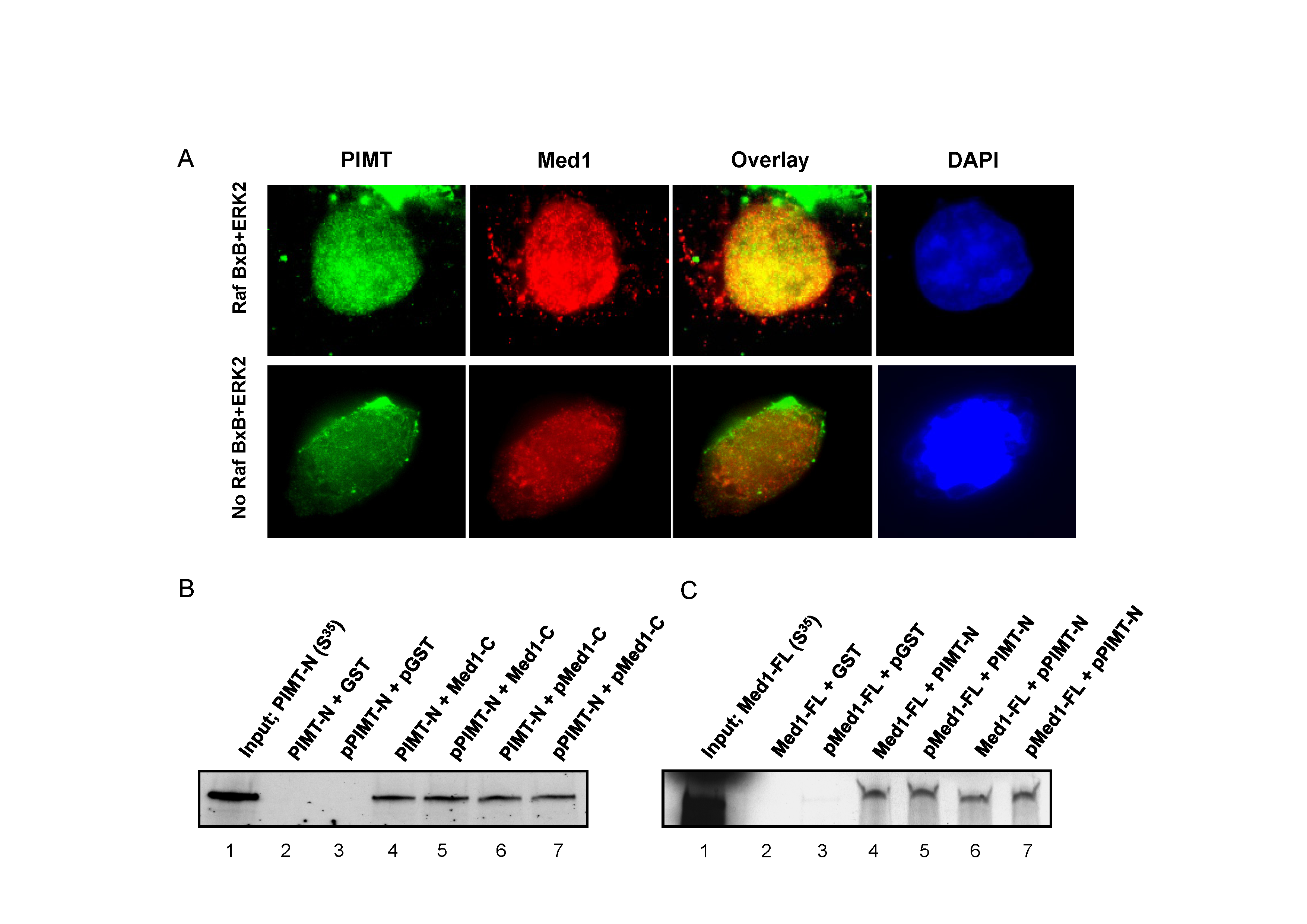

Supplement: Figure S5 — PIMT- Med1 interaction is independent of their phosphorylation state (A) HeLa cells were transfected with Flag-tagged PIMT and Med1 along with or without Raf-BXB and ERK2. Nuclei were stained by DAPI (blue). FITC labeled anti FLAG and Cy3 labeled secondary antibody against Med1 were used to visualize the localization of PIMT and Med1, respectively using Deltavision deconvulation microscopy. (B) 35S labeled in vitro translated PIMT-N was and incubated for 2 h at 4°C with GST-Med1-C either unmodified or phosphorylated by purified ERK2. Following GST pull-down samples were resolved by SDS-PAGE and signals were visualized by autoradiography. (C) In vitro translated 35S labeled full length Med1 (Med1-FL) was incubated for 2 h at 4°C with GST-PIMT-N either unmodified or phosphorylated by purified ERK2. After incubation, PIMT-N was precipitated using glutathione sepharose beads followed by SDS-PAGE and autoradiography. (TIF) [file pone.0083787.s005.tif]
