# Supplementary material for: ERK2-Mediated Phosphorylation of Transcriptional Coactivator Binding Protein PIMT/NCoA6IP at Ser298 Augments Hepatic Gluconeogenesis
Source: PLoS One. 2013 Dec 17;8(12):e83787. doi: 10.1371/journal.pone.0083787 (PMC3866170; doi:10.1371/journal.pone.0083787)
Supplement: Methods S1 — Supporting Materials and Methods. (DOCX) [file pone.0083787.s006.docx]

**Materials and Methods**

**GST pull down assay**

For in vitro GST pull down assay, full length Med1 and PIMT-N (1-334) were labeled with 35S-methionine (Perkin Elmer, Wellesley, MA, USA) using in vitro transcription-translation coupled system (Promega, Madison, WI, USA). GST- Med1-C (1371-1560) or GST-PIMT-N (1-334) were phosphorylated in the presence of cold ATP using purified ERK1/ERK2 (Merck, Rahway, NJ, USA) and pull down was performed as described previously (1).

**Co-localization and deconvolution microscopy**

The assay was performed as reported earlier (1). In brief, HeLa cells were transfected with pCMV-PIMT-Flag along with pcDNA3.1-Med1 using Lipofectamine 2000 (Invitrogen, Carlsbad, CA, USA according to manufacturer’s recommendations. Cells were incubated with FITC labeled anti FLAG antibody (F4049, Sigma, St. Louis, MO, USA) or anti- TRAP220/PBP (sc-5334, Santacruz biotechnology Inc. Santacruz,, CA, USA), followed by incubation with secondary antibody (Donkey-anti-Goat Alexa fluor; A21447, Invitrogen, Carlsbad, CA, USA) to visualize PIMT and Med1, respectively. The nucleus was stained using DAPI. Fluorescence microscopy and digital image collection were performed by using Olympus microscope equipped with photometrix cooled charge-coupled device camera driven by DELTAVISION software from Applied Precision (Seattle, WA, USA).

1. Misra, P., Owuor, E. D., Li, W., Yu, S., Qi, C et al. (2002) Phosphorylation of transcriptional coactivator peroxisome proliferator activated receptor (PPAR)-binding protein (PBP). Stimulation of transcriptional regulation by mitogen-activated protein kinase. *The Journal of Biological Chemistry* **277**, 48745-48754
