# Supplementary material for: ERK2-Mediated Phosphorylation of Transcriptional Coactivator Binding Protein PIMT/NCoA6IP at Ser298 Augments Hepatic Gluconeogenesis
Source: PLoS One. 2013 Dec 17;8(12):e83787. doi: 10.1371/journal.pone.0083787 (PMC3866170; doi:10.1371/journal.pone.0083787)
Supplement: Table S1 — Primer sequences. Sequences of the primers used in this study are provided. Nucleotides in bold represent mutations and primers prefixed with letter “Q” were used for qPCR analysis. (DOCX) [file pone.0083787.s007.docx]

**Table S1: Primer sequences**

| **hPEPCK promoter** | **Fwd**: GACGAGCTCTAGGACTCCAAATGAGTTCTG |
| --- | --- |
|  | **Rev**: AGGCTCGAGCTCTTTGGATGATCTCGAAG |
| **hPIMT^S298A^** | **Fwd**: TAGTATCTTTTCCATCT**GCA**CCTATTATGGTTGATAATG |
|  | **Rev**: CATTATCAACCATAATAGG**TGC**AGATGGAAAAGATACTA |
| **hPIMT^S298D^** | **Fwd**: TAGTATCTTTTCCATCT**GAC**CCTATTATGGTTGATAATG |
|  | **Rev**: CATTATCAACCATAATAGG**GTC**AGATGGAAAAGATACTA |
| **Q-rPEPCK** | **Fwd**: TGCTGGTGTCCCTCTAGTCTATGAA |
|  | **Rev**: AGCCAGTGGGCCAGGTATTT |
| **Q-mPEPCK** | **Fwd:** TGAACTGACAGACTCGCCCT |
|  | **Rev:**  GTCTTCCCACAGGCACTAGG |
| **Q-rG6pase** | **Fwd:** TCCACCTTGACACTACACCC |
|  | **Rev:** GGGACGGTCGCACTCTT |
| **Q-rPGC1α** | **Fwd:** ATGAATGCAGCGGTCTTAGC |
|  | **Rev:** AACAATGGCAGGGTTTGTTC |
| **Q-rHNF4α** | **Fwd:** GGTGCCAACCTCAACTC |
|  | **Rev:** CGCTCCTCCTGAAGAAT |
| **Q-hPIMT** | **Fwd**: CTGGAGGGCAAGTGGAAATA |
|  | **Rev**: GTTTCAGAGGCTGGTCTTCG |
| **Q-mMed1** | **Fwd**: GAGACTCCGCCCACTTACCG |
|  | **Rev**: GGACATTCAACTGGAGG |
| **Q-18S** | **Fwd**: AAACGGCTACCACATCCAAG |
|  | **Rev**: CCTCCAATGGATCCTCGTTA |
| **Q-rGAPDH** | **Fwd:** ACAGCAACAGAGTGGTGGAC |
|  | **Rev:** TTTGAGGGTGCAGCGAACTT |
| **ChIP rPEPCK** | **Fwd:** AGAGGATCCAGCAGACACCTAGT |
|  | **Rev:** CCAGTGGCTGCTGGTTGTCAA |
| **ChIP hPEPCK TRE-GRE** | **Fwd:** CCAACCAGCAGCTCTTGGT |
|  | **Rev:** ACCCATTTTACTGCTGTTGCAAAAC |
| **ChIP hPEPCK PPRE** | **Fwd:** TCCAAACTCCAGCAAGCAGCT |
|  | **Rev:** TCCCTGGCAAGTCTTAGAGTTTAGG |
